# Supplementary material for: Identifying the top research priorities in medically not yet explained symptoms (MNYES): a James Lind Alliance priority setting partnership
Source: BMJ Open. 2022 Jul 1;12(7):e061263. doi: 10.1136/bmjopen-2022-061263 (PMC9252198; doi:10.1136/bmjopen-2022-061263)
Supplement: Supplementary data [file bmjopen-2022-061263supp001.pdf]

Appendix

Key words included in evidence check searches

**Symptom terms**	**Publication focus**
Medically not yet explained symptoms	Guideline
Medically unexplained symptoms (MUS)	Mechanism
Persistent Somatic Symptoms	Pathology
Somatic Symptom and Related Disorders (SSRD)	Development
Chronic pain	Assessment
Unexplained pain	Diagnosis
Fatigue	Investigation
Unexplained fatigue	Treatment
Conversion disorder	Management
Neurological conversion symptoms	Healthcare service
Functional neurological disorder	Patient care
Functional symptoms	Care pathway
Functional neurological symptom	Outcome
Functional cognitive symptom	Prognosis
Functional weakness	
Dissociative disorders	
Dissociative neurological disorder	
Dissociative syncope	
Unexplained dizziness	
Fibromyalgia	
Irritable bowel syndrome (IBS)	
Nonepileptic seizures	

Acquiring funding, international aspect, and establishing support by the James Lind Alliance

Funding for the study was acquired by the PSP lead (CFC) in November 2019. Part of the collaboration was envisioned to support setting up a parallel PSP Steering Group to explore the same question in the Netherlands, in order to have an international perspective. This article focuses on the description of the process in the UK. Support by the James Lind Alliance Priority Setting Partnership was secured in December 2019 and a JLA Advisor (JG) appointed to the project.

Box 1: Steering group composition

Clinicians: One general practitioner(GP); consultants in cardiology (1), neurology (1), endocrinology (1), clinical liaison psychiatry (3) and rheumatology (1) one stroke physician, a clinical psychologist and one physiotherapist.

Patients and caregivers: Four patients with a variety of pain, fatigue and functional neurological symptoms; one caregiver;

Other: Two information specialists and two project coordinators to facilitate the PSP working group. They prepared meeting documents, surveys, supported recruitment, completed evidence checking and analysis but did not engage in the priority setting.

The consultative process

The study followed several steps in order to establish research priorities, using a mix of online surveys and a priority setting workshop. We created a website to host the surveys (27) and sent out the website link for our partner organisations to distribute within their networks.

Gathering uncertainties

The initial survey was launched between June 2020 and January 2021 and asked patients, caregivers and healthcare professionals to indicate their priorities for future research related to MNYES (27). We promoted the survey through partner organisations' websites, and their social media platforms. In addition, the clinics of clinicians involved in the PSP Steering Group distributed the QR code to their patients. We sent out email reminders to members of professional associations, sent Twitter invitations and placed the survey on the University of York and JLA websites. Distribution of the survey was supported by the members of the PSP, several associations, networks, networking groups and charities. An overview of supporting

organisations is shown in Table 1, below. Some of them collaborated because they found that patients with unexplained symptoms often visit their websites and related specialists to assess their symptoms.

Table 1: Supporting organisations who have promoted this work

**Charities**	**Professional networks**	**Other networks and Social media groups**
British Thyroid Foundation	Allied Health Professional FND Networking groups	INCLUDE (University of York Disability Network)
Fibromyalgia Association UK	Clinical special interest groups	James Lind Alliance
FND Hope	Liaison psychiatry networks	People who have shared information with their friends and family
Graham Hughes International	Royal College of Psychiatrists	Individuals and organisations active on twitter
Guts UK		Special interest and support groups on facebook
Health Unlocked		
Pain UK		
POTS-UK		

We targeted patients with MNYES such as pain, bowel problems, functional neurlogical symptoms, or with diagnoses which comprise a set of symptoms which are medically unexplained such as postural orthostatic tachycardia syndrome (PoTS); their caregivers; and the clinicians who treat them, to participate. Survey respondents were asked whether they had experienced MYNES, in which case they were to select their symptoms from a list, or were healthcare professionals, in which case they were asked to state their profession. Anonymity was preserved in all cases accordance with General Data Protection regulations.

Data analysis and verifying uncertainties

We downloaded the online survey results through Qualtrics and exported them into Microsoft Excel. The responses to this survey were organised into summary questions and these were then checked to make sure that they had not already been answered by research.(16) Our health information specialists (DV and JS) and PSP lead (CFC) grouped the responses into themes and each was analysed in small groups by 3 or 4 members of the Steering Group; one member of each small group was a patient or caregiver and the other members were healthcare professionals. Summary questions were developed, which encapsulated groupings of similar responses to the survey. Responses were removed if they were outside the scope

of the survey or general statements which would be unanswerable by research. The outputs from the small group discussions were reviewed at a subsequent meeting of the full PSP Steering Group.

One health information specialist (JS) checked each in-scope question with existing clinical guidelines published by multi-disciplinary guideline committees and systematic reviews, to ensure that the question had not been already fully answered with high-level evidence. 44 articles were shortlisted from searches conducted in the Cochrane Library, Medline and Google Scholar; key words for the search used are included in the appendix. Reference lists of these studies were also reviewed to identify further relevant references. Articles published between 2011 and 2021 were considered relevant by the Steering Group. Each question was coded to indicate whether it was 'answered'; 'partially answered' or 'unanswered' through the research literature.

Interim priority setting

Between May and July 2021, the second online survey asked participants to select their top 10 priorities from the list of uncertainties presented (19). The second survey was launched independently from the first survey. Questions were presented in a random order to each survey participant to reduce bias. The priorities of patients and caregivers and the priorities from healthcare professionals were ranked separately. The top 10 priorities identified by patients and caregivers were then combined with the top 10 priorities from healthcare professionals to create a shortlist of 17 priorities as there were 3 overlapping priorities.

Final workshop

The 17 top ranked questions were taken forward to the final priority setting consensus meeting that took place virtually on 16th and 17th September 2021. We invited volunteers from supporting organisations, two of whom joined the meeting. We also invited individuals who took part in the online survey to participate in the meeting. 25 participants took part in the workshop comprising 10 patients with a variety of MNYES symptoms, 1 caregiver, and 14 health care professionals. The workshop participants were divided into four groups, each with a balanced mix of patients/caregivers and clinicians. Each group also had observers who did not participate in the process, to fulfill the requirement of transparency of the JLA process. Each group was asked to rank the uncertainties through group discussions using the modified

nominal group technique facilitated by an independent JLA Advisor. Each participant was provided with a printed and electronic copy of the different 'unanswered' questions, and the JLA Advisor leading each group shared an image containing the unanswered questions to aid the group in ranking the 17 uncertainties. The rankings from the four groups were combined and all the workshop participants then came together to discuss these rankings. Similarities and differences between the group rankings were highlighted by the JLA facilitator leading the workshop (JG) and participants were invited to comment on the initial combined ranking. Following this, participants were reallocated into four new groups, maintaining the representation of patients/caregivers and clinicians within each group, to consider the combined group ranking from the previous session. During this session, there was a specific focus placed on the ordering of uncertainties across the top 17 unanswered questions. The rankings from each of the four groups were once again combined and were presented to the whole group for discussion. By the end of the priority setting meeting, we reached consensus on the top 10 UK research priorities for MNYES.

The respondents were 77% female, 86% white; data from the 2011 census show that 51% of the England and Wales population were female (28) and 86% of the same population were White (29). Of the 884 statements, 852 were in scope and 32 out-of-scope. The out-of-scope submissions included general statements (N = 11), children/paediatric services (N = 9), information seeking statements (N = 6), or pertaining to COVID-19 (N = 5) and chronic fatigue syndrome (N = 1).

Table 2: Participant demographic information

	**Initial Survey** (n=443)	**Interim Survey** (n=270)	**Final Workshop** (n=25)***
**Gender (%)**			
Male	89 (20)	33 (12)	6 (24)
Female	339 (77)	227 (84)	18 (72)
Other	14*(3)	2** (1)	1 (4)
**Ethnicity (%)**			
Asian/ Asian British	12 (3)	5 (2)	-
Arab	1 (<1)	-	-
Black/ Black British	3 (1)	2 (1)	-
Mixed/ Multiple	7 (2)	7 (2)	-
White	381 (86)	242 (90)	25 (100)
Other	17 (4)	4 (1)	-
**Age**, mean (SD)	47.65 (12.15)	42.55 (13.26)	39.29 (13.74)
**Experience (%)**			
Patient	288 (65)	183 (68)	10 (40)

Caregiver	13 (3)	17 (6)	1 (4)
Clinician	121 (27)	65 (24)	14 (56)
Other	21 (5)	5 (2)	-
**MNYES symptoms as self-described(%)**			
Pain	219 (49)	147 (54)	6 (24)
Fatigue	236 (53)	146 (54)	8 (32)
Dizziness	156 (35)	91 (34)	5 (20)
Heart palpitations/fainting	134 (30)	83 (31)	5 (20)
Stomach/bowel problems	197 (44)	119 (44)	5 (20)
Problems with movement (tremors, shakes, weakness)	133 (30)	114 (42)	6 (24)
Unexplained blackouts, seizures, sudden loss of awareness	54 (12)	60 (22)	3 (12)
Other (e.g. brain fog, eye and skin problems)	126 (28)	44 (16)	4 (16)
**Age when first developed MNYES**, mean (SD)	31.77 (15.08)	29.89 (15.13)	-
**Clinical role as self-described**			
Cardiologist	-	1 (<1)	-
Consultant in emergency medicine	-	1 (<1)	-
Doctor	10 (2)	2 (1)	-
Gastroenterologist	-	-	1 (4)
GP	4 (1)	5 (2)	3 (12)
Occupational therapist	9 (2)	6 (2)	1 (4)
Neurologist	9 (2)	3 (1)	1 (4)
Neuropsychiatrist	1 (<1)	1 (<1)	-
Neuropsychologist	-	1 (<1)	-
Nurse	3 (1)	2 (1)	-
Physiotherapist	16 (4)	8 (3)	2 (8)
Psychiatrist	44 (10)	18 (7)	4 (16)
Psychologist	9 (2)	5 (2)	1 (4)
Psychotherapist	3 (1)	2 (1)	-
Other	8 (2)	1 (1)	-
Rheumatologist	1 (<1)	1 (<1)	
Speech and language therapist	1 (<1)	4 (1)	-
Stroke specialist	-	-	1 (4)
Assistant psychologist/Support worker	3 (1)	2 (1)	-

*14 prefer not to say **1 non-binary, Not mandatory and therefore not always provided

Figure S1 shows the proportion of original questions from each topic, which were posed by patients, caregivers, clinicians and others, who were generally individuals who met the criteria for more than one role. As can be seen, patients preferred aetiology and diagnostic questions, while clinicians emphasized treatment and outcomes. Health care services and prognosis were divided equally between patients or caregivers and clinicians.

Figure S1: The proportion of questions per domain suggested by patients, caregivers, clinicians and other

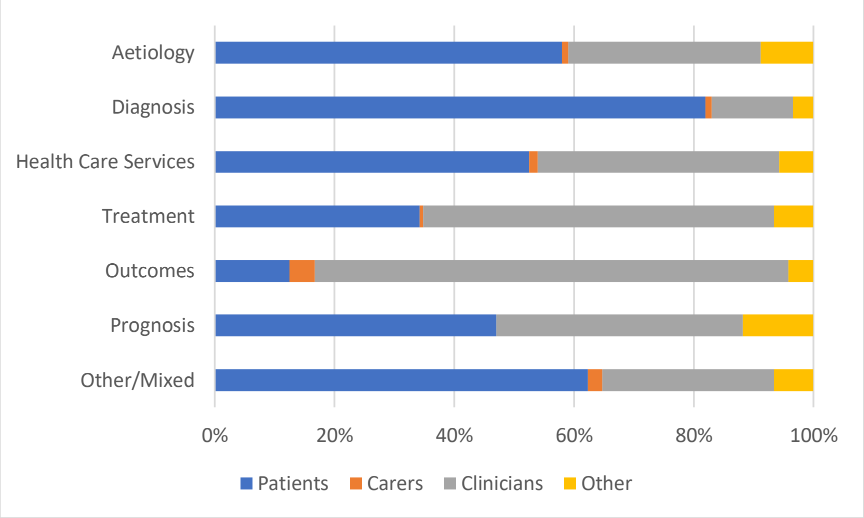

227 of the 264 (86%) people who reported gender were female and 242 of the 260 (93.1%) people who reported ethnicity, were white. The 17 resulting priorities were shortlisted for consideration at the final priority setting consensus workshop.

Box 2: research priority 11 through 17.

11. What is the relationship between mental health problems and MNYES?
12. What is the relationship over time between MNYES and known medical conditions and does that suggest some shared pathological process?
13. What is the best practice to offer optimal care for patients with MNYES?
14. What are current clinical attitudes and levels of knowledge about MNYES?
15. What are the most effective physical treatments for different symptoms of MNYES?
16. What are the most effective psychological treatments for different symptoms of MNYES?
17. Why do symptoms of MNYES fluctuate?
