# Supplementary material for: Identifying the top research priorities in medically not yet explained symptoms (MNYES): a James Lind Alliance priority setting partnership
Source: BMJ Open. 2022 Jul 1;12(7):e061263. doi: 10.1136/bmjopen-2022-061263 (PMC9252198; doi:10.1136/bmjopen-2022-061263)
Supplement: Author's manuscript [file bmjopen-2022-061263.draft_revisions.pdf]

BMJ Open is committed to open peer review. As part of this commitment we make the peer review history of every article we publish publicly available.

When an article is published we post the peer reviewers' comments and the authors' responses online. We also post the versions of the paper that were used during peer review. These are the versions that the peer review comments apply to.

The versions of the paper that follow are the versions that were submitted during the peer review process. They are not the versions of record or the final published versions. They should not be cited or distributed as the published version of this manuscript.

BMJ Open is an open access journal and the full, final, typeset and author-corrected version of record of the manuscript is available on our site with no access controls, subscription charges or pay-per-view fees (http://bmjopen.bmj.com).

If you have any questions on BMJ Open's open peer review process please email info.bmjopen@bmj.com

BMJ Open

Identifying the top research priorities in medically not yet explained symptoms (MNYES): a James Lind Alliance priority setting partnership

Journal:	*BMJ Open*
Manuscript ID	bmjopen-2022-061263
Article Type:	Original research
Date Submitted by the Author:	21-Jan-2022
Complete List of Authors:	van der Feltz-Cornelis, Christina; University of York, Dept. of Health Sciences, HYMS; Tees Esk and Wear Valleys NHS Foundation Trust<br>Sweetman, Jennifer; University of York<br>Edwards, Mark; St George's University of London; St George’s University Hospitals NHS Foundation Trust<br>Gall, Nicholas; University of London Kings College Hospital, Department of Cardiology<br>Gilligan, Jennifer; Tees Esk and Wear Valleys NHS Foundation Trust<br>Hayle, Steph; Patient representative<br>Kaul, Arvind; St George’s University Hospitals NHS Foundation Trust<br>Moriarty, Andrew; University of York Department of Health Sciences; Hull York Medical School,<br>Perros, Petros; Royal Victoria Infirmary, Department of Endocrinology<br>Sampford, James; Tees Esk and Wear Valleys NHS Foundation Trust<br>Smith, Natalie; University of York<br>Elfeddali, Iman; Tilburg University, Tranzo Dept.; GGz Breburg, Tilburg, the Netherlands, Centre for Body Mind and Health<br>Varley, Danielle; University of York Department of Health Sciences<br>Gower, Jonathan; JLA
Keywords:	Adult psychiatry < PSYCHIATRY, Adult cardiology < CARDIOLOGY, Functional bowel disorders < GASTROENTEROLOGY, Rheumatology < INTERNAL MEDICINE, Adult neurology < NEUROLOGY, PRIMARY CARE

SCHOLARONE™
Manuscripts

I, the Submitting Author has the right to grant and does grant on behalf of all authors of the Work (as defined in the below author licence), an exclusive licence and/or a non-exclusive licence for contributions from authors who are: i) UK Crown employees; ii) where BMJ has agreed a CC-BY licence shall apply, and/or iii) in accordance with the terms applicable for US Federal Government officers or employees acting as part of their official duties; on a worldwide, perpetual, irrevocable, royalty-free basis to BMJ Publishing Group Ltd ("BMJ") its licensees and where the relevant Journal is co-owned by BMJ to the co-owners of the Journal, to publish the Work in this journal and any other BMJ products and to exploit all rights, as set out in our licence.

The Submitting Author accepts and understands that any supply made under these terms is made by BMJ to the Submitting Author unless you are acting as an employee on behalf of your employer or a postgraduate student of an affiliated institution which is paying any applicable article publishing charge ("APC") for Open Access articles. Where the Submitting Author wishes to make the Work available on an Open Access basis (and intends to pay the relevant APC), the terms of reuse of such Open Access shall be governed by a Creative Commons licence – details of these licences and which Creative Commons licence will apply to this Work are set out in our licence referred to above.

Other than as permitted in any relevant BMJ Author's Self Archiving Policies, I confirm this Work has not been accepted for publication elsewhere, is not being considered for publication elsewhere and does not duplicate material already published. I confirm all authors consent to publication of this Work and authorise the granting of this licence.

For peer review only

Identifying the top research priorities in medically not yet explained symptoms (MNYES): a James Lind Alliance priority setting partnership

Christina M van der Feltz-Cornelis,[1,2,3] Jennifer F Sweetman,[1] Mark Edwards,[4,5] Nicholas Gall,[6] Jennifer Gilligan,[3] Steph Hayle,[7] Arvind Kaul,[5] Andrew S Moriarty,[1,2] Petros Perros,[8] James Sampford,[3] Natalie Smith,[1] Iman Elfeddali,[9,10] Danielle Varley,[1] Jonathan Gower[11]

Corresponding author: Christina van der Feltz-Cornelis, Dept. of Health Sciences, HYMS, University of York, ARRC Building, T204, Heslington, York, United Kingdom. Phone +441904321313 Email christina.vanderfeltz-cornelis@york.ac.uk

[1] Dept of Health Sciences, University of York, York, United Kingdom

[2] Hull York Medical School, (HYMS), University of York, York, United Kingdom

[3] Tees Esk and Wear Valley NHS Foundation Trust, Darlington, United Kingdom

[4] Neuroscience Research Centre, Institute of Molecular and Clinical Sciences, St George's University of London, London, United Kingdom.

[5] St. George's University Hospitals NHS Foundation Trust, London, United Kingdom

[6] Department of Cardiology, King's College Hospital, Denmark Hill, London, SE5 9RS, United Kingdom

[7] Patient representative, North Yorkshire, United Kingdom

[8] Department of Endocrinology, Royal Victoria Infirmary, Newcastle upon Tyne, United Kingdom

[9] Tranzo Dept., Tilburg University, Tilburg, the Netherlands

[10] Centre for Body Mind and Health, GGz Breburg, Tilburg, the Netherlands

[11] James Lind Alliance, Southampton, United Kingdom

For peer review only - http://bmjopen.bmj.com/site/about/guidelines.xhtml

Abstract

Objectives Research into medical symptoms that cannot be explained by known medical conditions, termed Medically Not Yet Explained Symptoms (MNYES), in general has been poorly funded over the years. However, such symptoms lead to distress and high disability, both individually and societally. Patients often cannot work and depend on family for care. Research so far has been primarily researcher-led with minimal input from patients, caregivers and clinicians; and sometimes has been controversial. Hence we brought together patients, caregivers and clinicians in a priority setting partnership (PSP) to identify the most important unanswered research questions in MNYES from their perspectives, following the James Lind Alliance (JLA) approach.

Design Delphi study according to the JLA priority setting method.

Methods The study involved five key stages: defining the appropriate term for the conditions under study by the PSP Steering Group; gathering questions on MNYES from patients, caregivers and clinicians in a publicly accessible survey; checking these research questions against existing evidence; interim prioritisation in a second survey; and a final multi-stakeholder consensus meeting to determine the top 10 unanswered research questions using the modified nominal group methodology.

Results Over 700 responses from UK patients, caregivers and clinicians were identified in the two surveys and charities contributed from a broad range of medical specialties and primary care. The final top 10 unanswered research questions cover, amongst others: treatment strategies, collaborative care pathways, training for clinicians, outcomes that matter to patients, and personalisation of treatment.

Interpretation The top 10 unanswered research questions are expected to generate much needed, relevant and impactful research into MNYES.

Word count: 4375

Key words: James Lind Alliance; Priority Setting; Medically Not Yet Explained Symptoms (MNYES); Medically Unexplained Symptoms (MUS); Delphi study

For peer review only - http://bmjopen.bmj.com/site/about/guidelines.xhtml

Article summary

- This study establishes research priorities for medically not yet explained symptoms (MNYES) from the perspective of patients, caregivers and clinicians.
- The term MNYES was chosen by the PSP Steering Group as an operational definition not intended to add to or replace other definitions already in use, but was constructed to embrace the views of all stakeholders for the duration of the study.

Strengths and limitations of this study

- Use of established and transparent James Lind Alliance methodology.
- Over 700 responses were gathered from patients, caregivers and clinicians from the whole array of medical specialties including primary care, indicating that the priorities were widely supported.
- The contributions of people from ethnic and gender minority groups within the PSP steering group, surveys and final workshop supports the inclusive nature of this work and indicates these priorities are important to a diverse range of people.

For peer review only - http://bmjopen.bmj.com/site/about/guidelines.xhtml

Introduction

Medically Not Yet Explained Symptoms (MNYES), represent up to 30% of presentations in primary care and internal medicine settings (1-3). They can include fatigue, pain, dizziness, irritable bowel syndrome, and functional neurological symptoms (FND)(3). They are often deemed to be stress-related, or of psychological origin, and comorbid depressive or anxiety disorder occur in approximately 30% of cases (2). Patients diagnosed with these symptoms often feel that they are not taken seriously, although care may have been taken to explain their condition properly. It takes too long to reach the conclusion that they have MNYES, whereas they typically experience high disability and visit many clinics for diagnostic procedures to hear no cause can be found for their symptoms, from clinicians who often have a dualistic view of health and disease. This inevitably leads to disappointment and frustration (4). Many clinicians lack confidence in the assessment and management of MNYES, or may exhibit behaviours perceived as dismissive. Patients often perceive a stigmatising attitude from clinicians and a sense that they are being judged as neurotic or mentally unwell (5-7). Moreover, management plans may not be sufficiently holistic to address all patient concerns, and effective treatments are scarce. All the above impact negatively on long-term prognosis.

The focus of research on MNYES is often on particular subsets of symptoms, such as chronic pain, chronic fatigue, irritable bowel syndrome, and dizziness, but lacks a comprehensive view. This has ramifications for patients who visit different clinics for their various symptoms, without sustained improvement, and as such experience unmet needs (8, 9). To address this, the University of York through the lead author (CFC) established a Priority Setting Partnership (PSP) for research needed to address MNYES. We engaged with members of the public, patients with MYNES and their carers, clinicians of all medical specialties known to have patients with MNYES (10), and other key stakeholders such as charities and the Royal College of Psychiatry Liaison Faculty. Close collaboration with the James Lind Alliance (JLA) enabled this PSP to follow their established, rigorous approach to identify the treatment and management priorities of stakeholders (patients, caregivers, clinicians and support organisations) and to incorporate these into a research agenda (11).

The European Association of Psychosomatic Medicine has published a research agenda in this domain with one of the research priorities being patient preferences for research in this field

For peer review only - http://bmjopen.bmj.com/site/about/guidelines.xhtml

(12). Until now, however, there has been relatively little support available for people with MNYES and those who care for them, to enable them in setting up the research agenda. Engaging patients in the research process incorporates their perspective as ‘experts’ from their unique experience of living with symptoms, as well as their personal knowledge regarding diagnostic trajectories and treatments in the healthcare setting if such symptoms remain (partially) unexplained (13). This study aims to address this knowledge gap.

The aim of this PSP was therefore to develop a research agenda with the joint perspectives of patients, caregivers, clinicians and support organisations across the United Kingdom as the frame of reference, to identify the most important unanswered research questions in MNYES.

Methods

Acquiring funding, international aspect, and establishing support by the James Lind Alliance

Funding for the study was acquired by the PSP lead (CFC) in November 2019. Part of the collaboration was envisioned to support setting up a parallel PSP Steering Group to explore the same question in the Netherlands, in order to have an international perspective. This article focuses on the description of the process in the UK. Support by the James Lind Alliance Priority Setting Partnership was secured in December 2019 and a JLA Advisor (JG) appointed to the project.

Establishing the Steering Group

In March 2020 the MNYES PSP Steering Group met for the first time. The Steering Group was tasked with overseeing the Priority Setting Partnership by having meetings every six weeks, chaired by the JLA advisor, and making critical decisions at key points of the project (14). The composition of the Steering Group is shown in Box 1 below.

Box 1: Steering group composition

Clinicians: One general practitioner(GP); consultants in cardiology (1), neurology (1), endocrinology (1), clinical liaison psychiatry (3) and rheumatology (1) one stroke physician, a clinical psychologist and one physiotherapist.

Patients and caregivers: Four patients with a variety of pain, fatigue and functional neurological symptoms; one caregiver;

Other: Two information specialists and two project coordinators.

Terminology

Many terms are used for these symptoms, as shown in a non-exhaustive list in Box 2, below. Therefore, the study's PSP Steering Group took time to decide what terminology to use in the study. A common concern appeared to be the distress caused to patients, caregivers and clinicians alike by the lack of adequate explanations, diagnostic methods and treatments for these symptoms – which are often poorly understood across these groups too. This was felt to have a negative impact on clinical work and research pertaining to these conditions and to stigmatise them at a societal level.

Box 2: Terminology

Persistent Somatic Symptoms (PSS)
Somatic Symptom and Related Disorders (SSRD) (15)
Bodily Distress Disorders (BDD) (16)
Medically Unexplained Symptoms (MUS)
Functional symptoms
Chronic pain
Conversion disorder (CD)
Dissociative neurological symptoms
Functional Neurological Disorder (FND)
Irritable Bowel Syndrome (IBS)
Dissociative syncope

After deliberation, the PSP Steering Group agreed to use the term *Medically Not Yet Explained Symptoms (MNYES)* to describe the subject matter for the duration of the study. This was an operational definition not intended to add to or replace other definitions already in use, that was constructed to embrace the views of all stakeholders. MNYES was meant to indicate that although some insights might exist, our understanding is still incomplete. This could pertain to biological, psychological and social factors, as well as factors involving the trajectory of patients through various healthcare settings. In that sense, the choice of the term MNYES conveys a message of hope, which feeds into the effort to identify research priorities for the condition. There is an ongoing debate amongst researchers and clinicians about how to refer

For peer review only - http://bmjopen.bmj.com/site/about/guidelines.xhtml

to these conditions. This may seem semantic, however it underpins the conceptual confusion that exists regarding these symptoms (17). Unfortunately, in some cases such uncertainty can give rise to deeply rooted controversy that ultimately can be traced back to lack of knowledge regarding the underlying conditions, and to related stigma. This knowledge gap could either be a factual lack of evidence, or a lack of availability of existing knowledge to clinicians, patients and the general public alike.

Inclusion and exclusion criteria

The PSP's Steering Group agreed that the remit should include the aetiology, diagnosis and treatment or medical care of patients with MNYES in the UK, as well as the organisation of services, social consequences and long term outcomes including cost implications for patients. Confirmed topics included (but were not limited to): pain, fatigue, dizziness, functional neurological disorder, bowel symptoms, palpitations and syncope. Ages 16 and older were included. Although fatigue as a symptom was considered for inclusion, Chronic Fatigue Syndrome was considered out of scope since there was another PSP addressing this.

The consultative process

The study followed several steps in order to establish research priorities, using a mix of online surveys and a priority setting workshop. We created a website to host the surveys (18) and sent out the website link for our partner organisations to distribute within their networks.

Gathering uncertainties

The initial survey was launched between June 2020 and January 2021 and asked patients, caregivers and healthcare professionals to indicate their priorities for future research related to MNYES (18). We promoted the survey through partner organisations' websites, and their social media platforms. In addition, the clinics of clinicians involved in the PSP Steering Group distributed the QR code to their patients. We sent out email reminders to members of professional associations, sent Twitter invitations and placed the survey on the University of York and JLA websites. Distribution of the survey was supported by the members of the PSP, several associations, networks, networking groups and charities. An overview of supporting organisations is shown in Table 1, below.

For peer review only - http://bmjopen.bmj.com/site/about/guidelines.xhtml

Table 1: Supporting organisations who have promoted this work

Charities	Professional networks	Other networks and Social media groups
British Thyroid Association	Allied Health Professional FND Networking groups	INCLUDE (University of York Disability Network)
Fibromyalgia Association UK	Clinical special interest groups	James Lind Alliance
FND Hope	Liaison psychiatry networks	People who have shared information with their friends and family
Graham Hughes International	Royal College of Psychiatrists	Individuals and organisations active on twitter
Guts UK		Special interest and support groups on facebook
Health Unlocked		
Pain UK		
POTS-UK		

We targeted patients with MNYES such as pain, bowel problems, functional neurlogical symptoms, or with diagnoses which comprise a set of symptoms which are medically unexplained such as postural orthostatic tachycardia syndrome (PoTS); their caregivers; and the clinicians who treat them, to participate. Survey respondents were asked whether they had experienced MYNES, in which case they were to select their symptoms from a list, or were healthcare professionals, in which case they were asked to state their profession. Anonymity was preserved in all cases accordance with General Data Protection regulations.

Data analysis and verifying uncertainties

We downloaded the online survey (11) results through Qualtrics and exported them into Microsoft Excel. The responses to this survey were organised into summary questions and these were then checked to make sure that they had not already been answered by research. Our health information specialists (DV and JS) and PSP lead (CFC) grouped the responses into themes and each was analysed in small groups by 3 or 4 members of the Steering Group; one member of each small group was a patient or caregiver and the other members were healthcare professionals. Summary questions were developed, which encapsulated groupings of similar responses to the survey. Responses were removed if they were outside the scope of the survey or general statements which would be unanswerable by research. The outputs from the small group discussions were reviewed at a subsequent meeting of the full PSP Steering Group.

For peer review only - http://bmjopen.bmj.com/site/about/guidelines.xhtml

One health information specialist (JS) checked each in-scope question with existing clinical guidelines published by multi-disciplinary guideline committees and systematic reviews, to ensure that the question had not been already fully answered with high-level evidence. 44 articles were shortlisted from searches conducted in the Cochrane Library, Medline and Google Scholar; key words for the search used are included in the appendix. Reference lists of these studies were also reviewed to identify further relevant references. Articles published between 2011 and 2021 were considered relevant by the Steering Group. Each question was coded to indicate whether it was 'answered'; 'partially answered' or 'unanswered' through the research literature. All of the questions fell within the latter two categories and were carried forward to the interim priority setting stage.

Interim priority setting

Between May and July 2021, the second online survey asked participants to select their top 10 priorities from the list of uncertainties presented (14). Questions were presented in a random order to each survey participant to reduce bias. The priorities of patients and caregivers and the priorities from healthcare professionals were ranked separately. The top 10 priorities identified by patients and caregivers were then combined with the top 10 priorities from healthcare professionals to create a shortlist of 17 priorities as there were 3 overlapping priorities.

Final workshop

The 17 top ranked questions were taken forward to the final priority setting consensus meeting that took place virtually on 16th and 17th September 2021. We invited volunteers from supporting organisations, two of whom joined the meeting. We also invited individuals who took part in the online survey to participate in the meeting. 25 participants took part in the workshop comprising 10 patients with a variety of MNYES symptoms, 1 caregiver, and 14 health care professionals. The workshop participants were divided into four groups, each with a balanced mix of patients/caregivers and clinicians. Each group also had observers who did not participate in the process, to fulfill the requirement of transparency of the JLA process. Each group was asked to rank the uncertainties through group discussions using the modified nominal group technique facilitated by an independent JLA Advisor. Each participant was provided with a printed and electronic copy of the different 'unanswered' questions, and the JLA Advisor leading each group shared an image containing the unanswered questions to aid

For peer review only - http://bmjopen.bmj.com/site/about/guidelines.xhtml

the group in ranking the 17 uncertainties. The rankings from the four groups were combined and all the workshop participants then came together to discuss these rankings. Similarities and differences between the group rankings were highlighted by the JLA facilitator leading the workshop (JG) and participants were invited to comment on the initial combined ranking. Following this, participants were reallocated into four new groups, maintaining the representation of patients/caregivers and clinicians within each group, to consider the combined group ranking from the previous session. During this session, there was a specific focus placed on the ordering of uncertainties across the top 17 unanswered questions. The rankings from each of the four groups were once again combined and were presented to the whole group for discussion. By the end of the priority setting meeting, we reached consensus on the top 10 UK research priorities for MNYES.

Patient and public involvement

A core principle of JLA priority setting partnerships is collaboration between all stakeholders (patients, caregivers, and clinicians) to ensure their views are represented at each stage of the process. Patients, caregivers and clinicians were members of the MNYES Steering Group, represented at every meeting, involved in the development of PSP surveys. They participated in the initial and interim surveys, and were involved in the organisation of uncertainties, the wording of summary questions, and the verification of evidence checking. The final workshop also included patients, caregivers and clinicians in the final prioritisation process to establish the top 10 research priorities for MNYES. Furthermore, there were observers representing supporting organisations and relevant charitable organisations during the final workshop. All Steering Group members were invited to contribute to the dissemination of the surveys; the information shared by this PSP was developed with members from all stakeholder groups. All PSP steering group members were invited to contribute to the article describing the findings and one of them indeed contributed as a co-author.

Results

The process is shown in the project flowdiagram presented in Figure 1 below.

For peer review only - http://bmjopen.bmj.com/site/about/guidelines.xhtml

- Insert **Figure 1. Flow diagram of MNYES question prioritisation processes** –

First survey

There were 705 respondents across the UK who accessed the initial survey. 443 of those people provided at least one question or statement within this survey. 68% of the participants were patients or caregivers as reported in Table 2 below, which also shows the demographic characteristics of the participants.

The respondents were 77% female, 86% white; data from the 2011 census show that 51% of the England and Wales population were female (19) and 86% of the same population were White (20).

Of the 884 statements, 852 were in scope and 32 out-of-scope.

The out-of-scope submissions included general statements (N = 11), children/paediatric services (N = 9), information seeking statements (N = 6), or pertaining to COVID-19 (N = 5) and chronic fatigue syndrome (N = 1).

The information specialists (DV and JS) and PSP lead (CFC), grouped similar or duplicate questions into 5 domains, generating 96 draft summary questions on aetiology, diagnosis, health care services, treatment, outcomes, prognosis, and other. Those 96 draft questions were reviewed by small groups of PSP Steering Group members that comprised clinicians, patients and caregivers. Further consolidations were made resulting in 46 summary questions which were reviewed again and signed off at a meeting of the whole PSP Steering Group. Of these 46 questions, 22% related to aetiology, 24% to health and clinical services, 15% to diagnosis, 24% to the treatment of MNYES, and 15% to outcomes.

For peer review only

For peer review only - http://bmjopen.bmj.com/site/about/guidelines.xhtml

Table 2: Participant demographic information

	**Initial Survey** (n=443)	**Interim Survey** (n=270)	**Final Workshop** (n=25)***
**Gender (%)**			
Male	89 (20)	33 (12)	6 (24)
Female	339 (77)	227 (84)	18 (72)
Other	14*(3)	2** (1)	1 (4)
**Ethnicity (%)**			
Asian/ Asian British	12 (3)	5 (2)	-
Arab	1 (<1)	-	-
Black/ Black British	3 (1)	2 (1)	-
Mixed/ Multiple	7 (2)	7 (2)	-
White	381 (86)	242 (90)	25 (100)
Other	17 (4)	4 (1)	-
**Age**, mean (SD)	47.65 (12.15)	42.55 (13.26)	39.29 (13.74)
**Experience (%)**			
Patient	288 (65)	183 (68)	10 (40)
Carer	13 (3)	17 (6)	1 (4)
Clinician	121 (27)	65 (24)	14 (56)
Other	21 (5)	5 (2)	-
**MNYES symptoms (%)**			
Pain	219 (49)	147 (54)	6 (24)
Fatigue	236 (53)	146 (54)	8 (32)
Dizziness	156 (35)	91 (34)	5 (20)
Heart palpitations/fainting	134 (30)	83 (31)	5 (20)
Stomach/bowel problems	197 (44)	119 (44)	5 (20)
Problems with movement (tremors, shakes, weakness)	133 (30)	114 (42)	6 (24)
Unexplained blackouts, seizures, sudden loss of awareness	54 (12)	60 (22)	3 (12)
Other (e.g. brain fog, eye and skin problems)	126 (28)	44 (16)	4 (16)
**Age when first developed MNYES**, mean (SD)	31.77 (15.08)	29.89 (15.13)	-
**Clinical role as self-described**			
Cardiologist	-	1 (<1)	-
Consultant in emergency medicine	-	1 (<1)	-
Doctor	10 (2)	2 (1)	-
Gastroenterologist	-	-	1 (4)
GP	4 (1)	5 (2)	3 (12)
Occupational therapist	9 (2)	6 (2)	1 (4)
Neurologist	9 (2)	3 (1)	1 (4)
Neuropsychiatrist	1 (<1)	1 (<1)	-
Neuropsychologist	-	1 (<1)	-
Nurse	3 (1)	2 (1)	-
Physiotherapist	16 (4)	8 (3)	2 (8)
Psychiatrist	44 (10)	18 (7)	4 (16)
Psychologist	9 (2)	5 (2)	1 (4)
Psychotherapist	3 (1)	2 (1)	-
Other	9 (2)	2 (1)	-
Speech and language therapist	1 (<1)	4 (1)	-
Stroke specialist	-	-	1 (4)
Assistant psychologist/Support worker	3 (1)	2 (1)	-

*14 prefer not to say **1 non-binary, and 1 self describing as agender

For peer review only - http://bmjopen.bmj.com/site/about/guidelines.xhtml

Figure 2 shows the proportion of original questions from each topic, which were posed by patients, caregivers, clinicians and others, who were generally individuals who met the criteria for more than one role. As can be seen, patients preferred aetiology and diagnostic questions, while clinicians emphasized treatment and outcomes. Health care services and prognosis were divided equally between patients or caregivers and clinicians.

- Insert **Figure 2 proportion of questions posed by patients, caregivers, clinicians and others**

Evidence check

As an evidence check, the 46 summary questions were checked against published systematic reviews and clinical guidelines. We found that none of the 46 summary questions had been fully answered by previous research; some questions had been answered for specific symptoms, but not comprehensively across all MNYES symptoms. At a subsequent meeting, the Steering Group reviewed the 46 summary questions in relation to the original questions and statements from which they derived. This process resulted in minor changes to the wording of these 46 questions which were then included in the interim prioritisation survey.

Interim survey

This online survey was completed by 270 participants from across the UK. Patients and caregivers made up 74% of the participants. Demographic information on the participants is shown in Table 2, however it was not mandatory for this survey and therefore not all respondents provided this information. 227 of the 264 (86%) people who reported gender were female and 242 of the 260 (93.1%) people who reported ethnicity, were white. The 17 resulting priorities were shortlisted for consideration at the final priority setting consensus workshop.

Final priority setting workshop

The final priority setting workshop was conducted remotely over two days. In total, 25 people participated in the workshop sessions; four JLA Advisors facilitated the subgroups, eight people observed and one person provided technical support. Participants included 11 people with MNYES or caregivers, and 14 healthcare professionals representing psychiatry, general practice, stroke, neurology, physiotherapy, psychology, occupational therapy and

For peer review only - http://bmjopen.bmj.com/site/about/guidelines.xhtml

gastroenterology. The final top 10 research priorities were agreed by consensus between all the participants as listed in Figure 3. They were placed on the James Lind Alliance Website (21). They are listed below:

- Insert **Figure 3. Top 10 research priorities** -

The research priorities which were ranked 11 – 17 are also listed on the JLA website and shown in Box 3:

Box 3: research priority 11 through 17.

11. What is the relationship between mental health problems and MNYES?
12. What is the relationship over time between MNYES and known medical conditions and does that suggest some shared pathological process?
13. What is the best practice to offer optimal care for patients with MNYES?
14. What are current clinical attitudes and levels of knowledge about MNYES?
15. What are the most effective physical treatments for different symptoms of MNYES?
16. What are the most effective psychological treatments for different symptoms of MNYES?
17. Why do symptoms of MNYES fluctuate?

Discussion

In this study, we used the JLA Priority Setting Partnership processes to identify the top 10 unanswered research questions for MNYES. We utilised the collective perspectives of patients, caregivers, and clinicians, and focused on areas where up-to-date, reliable research evidence is lacking. This process was supported by charitable and professional organisations across the United Kingdom. The study highlighted the paucity of evidence-driven practice in MNYES care since none of the 46 research questions gathered from our survey have been previously answered by level I evidence. Based on the extensive discussions that took place during the

For peer review only - http://bmjopen.bmj.com/site/about/guidelines.xhtml

meetings, the following major themes emerged from the top 10 unanswered research questions.

Theme 1: Treatment

The first and sixth research priorities concern treatment. The first, *What are the most effective treatment strategies for different symptoms of MNYES?* pertains to all potential treatment strategies (for example: pharmacological, psychological, physical, or collaboration models) to help manage or alleviate any MNYES or combination of symptoms, such as pain, fatigue, dizziness, functional neurological disorder, bowel symptoms, palpitations and syncope. The sixth research priority, *How can the most appropriate treatment be selected, dependent on different MNYES symptoms, that a person with MNYES is most likely to benefit from?* is about identifying the best ways to match people who experience specific MNYES with the treatments that are most likely to benefit them. This relates to personalisation of treatment based on diagnostic factors, such as a history of trauma, biomarkers or treatment needs. Given the high unmet clinical need and the heterogenity of MNYES, it is no surprise that this is considered to be an important research priority. Interestingly, this was also a research priority identified by the Delphi study conducted amongst experts in the field on behalf or the European Association of Psychosomatic Medicine (12); treatment and personalised treatment are therefore considered to be research priorities which are widely supported in this field.

Theme 2: The Role of Clinicians

Several research priorities involve the role of the clinicians in the diagnostic and treatment process. The second research priority, *How can clinicians collaborate effectively to form the most appropriate care pathway and service model to offer assessment and treatment for patients with MNYES?* pertains to the role that clinicians play towards each other. It includes the professionals and services which may be involved for the purpose of assessment, diagnosis and the provision or coordination of treatment. It relates to the fact that there are often many clinicians providing diagnostic assessments to people with MNYES, or are sought to provide treatment to them. This priority is focused on finding the best ways for clinicians to collaborate with each other to form an appropriate care pathway to support people with MNYES. This could be psychiatric consultation models, multi-disciplinary team models, collaborative care

For peer review only - http://bmjopen.bmj.com/site/about/guidelines.xhtml

models or other integrated care pathways. The third research priority, *What are the most effective methods for training clinicians to diagnose and treat their patients with MNYES with compassion, empathy and respect?* refers to medical practitioners who are already qualified and employed in positions that involve the diagnosis and treatment of people with MNYES. The focus of attention here is on the communication of information which acknowledges the perspective and concerns of the person experiencing MNYES. Priority five, *What are the most effective ways to support patients with MNYES and their carers to live with their symptoms?* focuses on identifying the range of different options for supporting people with MNYES and their caregivers. These may include but are not limited to: finding the most appropriate and acceptable ways to discuss MNYES with people who experience them and their caregivers; guidance about how to discuss MNYES with family members and friends; shared decision making when discussing treatment options; peer support organisations; reasonable adjustments in employment settings; adaptations to accommodation; psychological support to cope while living with symptoms; recovery and rehabilitation approaches. Another research priority fitting with this theme is priority nine: *What strategies are effective at promoting awareness and up to date clinical knowledge about MNYES amongst healthcare professionals?* This priority emphasises strategies to consistently and effectively ensure that clinicians are aware of and have the most up to date information about MNYES which will enable the care people receive to reflect current evidence. Given the existing knowledge gaps this is considered an important priority.

Theme 3: Symptoms and Outcomes

Patients were involved in all discussions regarding research priorities. However, some research priorities mention the patient perspective explicitly, such as, for example, priority four, *What outcomes matter most to patients with MNYES?* Based upon the survey answers, these may include, but are not limited to: symptom reduction; changes in biomarkers; improvements in abilities to undertake daily tasks; improvements in quality of life; individual goal achievements; or improvements in functioning. Priority seven, *What symptoms are commonly reported by people with MNYES and what links them?* relates to the fact that the list of MNYES is extensive and people who experience these symptoms often report living with multiple MNYES. The aim of this priority is to identify the symptoms which most commonly co-occur and the underlying factors and mechanisms between them. For priority eight, *What*

For peer review only - http://bmjopen.bmj.com/site/about/guidelines.xhtml

factors affect outcomes for MNYES? it was agreed the diversity of factors affecting outcomes should include biomarkers, psychological factors and trauma, health services-related issues such as feedback from recovered patients about what helped them, the availability and quality of information clinicians have access to, the way information is shared between clinicians and people experiencing MNYES, and social factors such as poverty, education, family dysfunction or domestic abuse, and work environment. Given the number of questions that pertained to aetiological factors, and the fact that the related uncertainty plays a role in the choice of MNYES as a term, this can be considered an important research theme.

Theme 4: Recovery

Research priority number ten relates to recovery: *Which self-management techniques are effective in MNYES?* this priority concerns the strategies that people experiencing MNYES can use separately from visits to clinics. The focus is to identify the most effective self-administered therapies for managing or alleviating MNYES, used separately, or in combination with formal treatment. Examples of self-management approaches include education, exercise, and dietary changes. It should be noted that this research priority, in contrast to ones covered by the other themes, considers that recovery in MNYES can occur, either by recovery of symptoms, or by personal recovery with ongoing symptomatology. Recovery of symptomatology is referred to as clinical recovery and is covered by the other themes. Recovery whilst symptoms are ongoing is called personal recovery (22), meaning that despite symptoms being present, function has to some extent been restored through treatment, self-management or disability management.

In mental health research and clinical practice, especially in relation to psychotic conditions, personal recovery is a construct that has increasingly gained attention over the past thirty years; however, so far the term has not been used in the context of MNYES. Generally, the emphasis, both in clinical practice and in research, seems to have been to either attempt to attain clinical recovery, or to send the patient home with the message that MNYES cannot be cured and that one would have to live with the condition. This dichotomy has fed into the ongoing controversy about how to approach MNYES. This polarising stance is unhelpful. Developing this research priority would require embracing the concept that personal recovery refers to an individual process of adaptation and development where one does not simply

For peer review only - http://bmjopen.bmj.com/site/about/guidelines.xhtml

return to, but rather grows beyond the premorbid self (23), with an emphasis on the patient perspective. It could provide an important contribution to the further development of research in this domain, alongside the research priorities summarised in the other themes.

Strengths of the study

This is the first study establishing research priorities for MNYES, according to the JLA Priority Setting Partnership method which incorporates the views of patients, caregivers and clinicians. This is a highly contentious area; however, the study outcomes were widely supported by over 400 participants including clinicians from a variety of disciplines, patients with a range of symptoms, caregivers, charitible organisations, and the Royal College of Psychiatry Liaison Faculty. The themes identified in this PSP cover a broad range of ideas, issues and uncertainties; these are outlined in the top 10 research priorities and also reflected in the seven research priorities that did not make the top 10. Research priorities 11, 12 and 17 would link well with theme 3 in the exploration of associations of MNYES with mental health and somatic comorbidity, as well as the development of symptoms over time. Priorities 13 and 14 would fit in theme two, the role of clinicians; 15 and 16 link with theme one, treatment. This suggests that the themes covered by the top 10 priorities are consistent with the rest of the research priorities which were proposed during this priority setting process.

Limitations of the study

The study provides a good overview of research priorities for MYNES in the UK, however given the specific cultural aspects and health care organisation in the UK, the findings may not be generalisable to other countries. As indicated earlier, a similar PSP is currently being conducted in the Netherlands and may shed light on research priorities in a non-NHS healthcare setting. This will give us an opportunity to compare and evaluate the generalisability of these findings and the influence of different cultural and healthcare settings. Future research highlighting the situation in Low and Middle Income Countries would be beneficial.

For peer review only - http://bmjopen.bmj.com/site/about/guidelines.xhtml

Conclusion

MYNES are common and reflect a high level of unmet clinical need. Incorporating patient-driven research in MNYES research can allow researchers to better address the complex care needs of people with MNYES. The most important aspect of this priority setting exercise was strengthening the relationship between patients, caregivers, clinicians and support organisations and generating a list of priorities valued by these stakeholders, which we hope will guide future research.

We have identified the top 10 research priorities in MNYES using the rigorous JLA priority setting method that may open the door to further research addressing the knowledge gaps and controversies in this area, and hopefully alleviate some of the stigma related to these conditions. Identification of these top 10 research priorities is an important first step to generating relevant and impactful research that will ultimately improve the lives of people with MNYES.

Acknowledgements: The steering group would like to thank to all the patients, carers, families, friends, healthcare professionals and supporting organisations who contributed to this work. In addition to the co-authors, Philippa Bolton, Sally Brabyn, Tracey Cunningham, Rosie Evans, Miriam Lomas, Margot Metz, Chris Price, Annie Shaw, Scott Spain, Lesley Spain were members of the PSP steering group. The following people who attended the final workshop for priority setting agreed to be named: Anna Burneika, Kit Byatt, Phoebe Cole, Tracey Cunningham, Mark Edwards, Rosie Evans, Eve Fazakerley, Jennifer Gilligan, Stephanie Johnston, Claire Jones, Hilary Lewis, Joseph Littlewood, Miriam Lomas, Andrew Moriarty, Elizabeth Paul, Emma Reinhold, Keziah Reiss, James Sampford, Annie Shaw, Gemma Smith, Martin Veysey, Juliet Wilson, Jennifer Wilson.

Ethics statement: Ethical approval was not required as confirmed by the research governance board of the Department of Health Sciences of the University of York.

For peer review only - http://bmjopen.bmj.com/site/about/guidelines.xhtml

Data availability statement

Data relevant to the study are included in the article or on the James Lind Alliance website (https://www.jla.nihr.ac.uk/priority-setting-partnerships/medically-not-yet-explained-symptoms/) and linked University of York website (https://www.york.ac.uk/healthsciences/research/mental-health/projects/mnyes/).

Conflicts of interest statement

The authors have no conflicts of interest to declare.

Author statement

CFC was the project lead, instigating the application to the JLA. CFC, NS, JFS and JG organised the steering group meetings. JFS and DV designed and built the surveys, analysed the data, and conducted the evidence check under supervision of JG and CFC. CFC, JFS, and NS wrote the majority of the final manuscript. JG (JLA Chair) chaired all the meetings, led the consensus workshop and ensured compliance with methodology throughout. NS and JFS took minutes for steering group meetings, built and distributed surveys, and organised the consensus workshop. JFS conducted the searches for the evidence check and screened the results with CFC. Members of the steering committee CFC, JFS, ME, NG, JG, SH, AK, ASM, PP, JS, NS, IE, DV, PB, SB, TC, RE, ML, MM, CP, AS, SS, LS all attended a majority of the meetings, agreed the initial protocol and the evidence check protocol, piloted and signed off the surveys and disseminated them, checked the raw questions against the indicative ones, reviewed the evidence check results and agreed the final longlist. TC, ME, JG, ML, ASM, JFS, and AS were present at the final consensus workshop. All authors reviewed and contributed to the final manuscript and approved it prior to submission.

Funding

This work was part-funded by the Wellcome Trust [ref: 204829] from the overall Wellcome Trust ISSF award through the Centre for Future Health (CFH) at the University of York.

References

1. Hoedeman R, Blankenstein AH, van der Feltz-Cornelis CM, Krol B, Stewart R, Groothoff JW. Consultation letters for medically unexplained physical symptoms in primary care. Cochrane Database of Systematic Reviews. 2010(12).
2. Van Hemert AM, Hengeveld MW, Bolk JH, Rooijmans HG, Vandenbroucke JP. Psychiatric disorders in relation to medical illness among patients of a general medical out-patient clinic. Psychological medicine. 1993;23(1):167-73.
3. Kirmayer LJ, Groleau D, Looper KJ, Dao MD. Explaining medically unexplained symptoms. The Canadian journal of psychiatry. 2004;49(10):663-72.
4. Salmon P, Peters S, Stanley I. Patients' perceptions of medical explanations for somatisation disorders: qualitative analysis. BmJ. 1999;318(7180):372-6.
5. Tolchin B, Tolchin DW, Stein MA. How Should Clinicians Minimize Harms and Maximize Benefits When Diagnosing and Treating Disorders Without Biomarkers? AMA journal of ethics. 2021;23(7):530-6.
6. Hatcher S, Arroll B. Assessment and management of medically unexplained symptoms. Bmj. 2008;336(7653):1124-8.
7. Stone J, Wojcik W, Durrance D, Carson A, Lewis S, MacKenzie L, et al. What should we say to patients with symptoms unexplained by disease? The “number needed to offend”. Bmj. 2002;325(7378):1449-50.
8. Carson AJ, Ringbauer B, Stone J, McKenzie L, Warlow C, Sharpe M. Do medically unexplained symptoms matter? A prospective cohort study of 300 new referrals to neurology outpatient clinics. Journal of Neurology, Neurosurgery & Psychiatry. 2000;68(2):207-10.
9. Reid S, Wessely S, Crayford T, Hotopf M. Frequent attenders with medically unexplained symptoms: service use and costs in secondary care. The British Journal of Psychiatry. 2002;180(3):248-53.
10. Nimnuan C, Hotopf M, Wessely S. Medically unexplained symptoms: an epidemiological study in seven specialities. Journal of psychosomatic research. 2001;51(1):361-7.
11. James Lind Alliance. The James Lind Alliance Guidebook Version 7. 2018.
12. van der Feltz-Cornelis CM, Elfeddali I, Werneke U, Malt UF, Van den Bergh O, Schaefert R, et al. A European research agenda for somatic symptom disorders, bodily distress disorders, and functional disorders: results of an estimate-talk-estimate Delphi expert study. Frontiers in psychiatry. 2018;9:151.
13. Gill PJ, Cartwright E. Partnering with patients in the production of evidence. Royal Society of Medicine; 2021.
14. James Lind Alliance. Priority Setting Partnership: Medically Not Yet Explained Symptoms 2021 [Available from: https://www.jla.nihr.ac.uk/priority-setting-partnerships/medically-not-yet-explained-symptoms/.
15. American Psychiatric Association. Diagnostic and statistical manual of mental disorders: DSM-5. Arlington, VA. 2013.
16. World Health Organization. International Classification of Diseases-11 (ICD-11)2019.
17. Van der Feltz-Cornelis C, Van Dyck R. The notion of somatization: an artefact of the conceptualization of body and mind. Psychotherapy and psychosomatics. 1997;66(3):117-27.
18. University of York. Research priority setting for Medically Not Yet Explained Symptoms (MNYES) in an Anglo-Dutch partnership with the James Lind Alliance 2021 [Available from: https://www.york.ac.uk/healthsciences/research/mental-health/projects/mnyes/.
19. GOV.UK. Male and female populations 2018 [Available from: https://www.ethnicity-facts-figures.service.gov.uk/uk-population-by-ethnicity/demographics/male-and-female-populations/latest.
20. GOV.UK. Population of England and Wales 2018 [Available from: https://www.ethnicity-facts-figures.service.gov.uk/uk-population-by-ethnicity/national-and-regional-populations/population-of-england-and-wales/latest.

For peer review only - http://bmjopen.bmj.com/site/about/guidelines.xhtml

21. James Lind Alliance. Medically Not Yet Explained Symptoms Top 10 priorities 2021 [Available from: https://www.jla.nihr.ac.uk/priority-setting-partnerships/medically-not-yet-explained-symptoms/top-10-priorities.htm.
22. Slade M, Amering M, Oades L. Recovery: an international perspective. Epidemiology and Psychiatric Sciences. 2008;17(2):128-37.
23. Slade M. Personal recovery and mental illness: A guide for mental health professionals: Cambridge University Press; 2009.

For peer review only

For peer review only - http://bmjopen.bmj.com/site/about/guidelines.xhtml

Figure 1: Flow diagram of MNYES question prioritisation processes

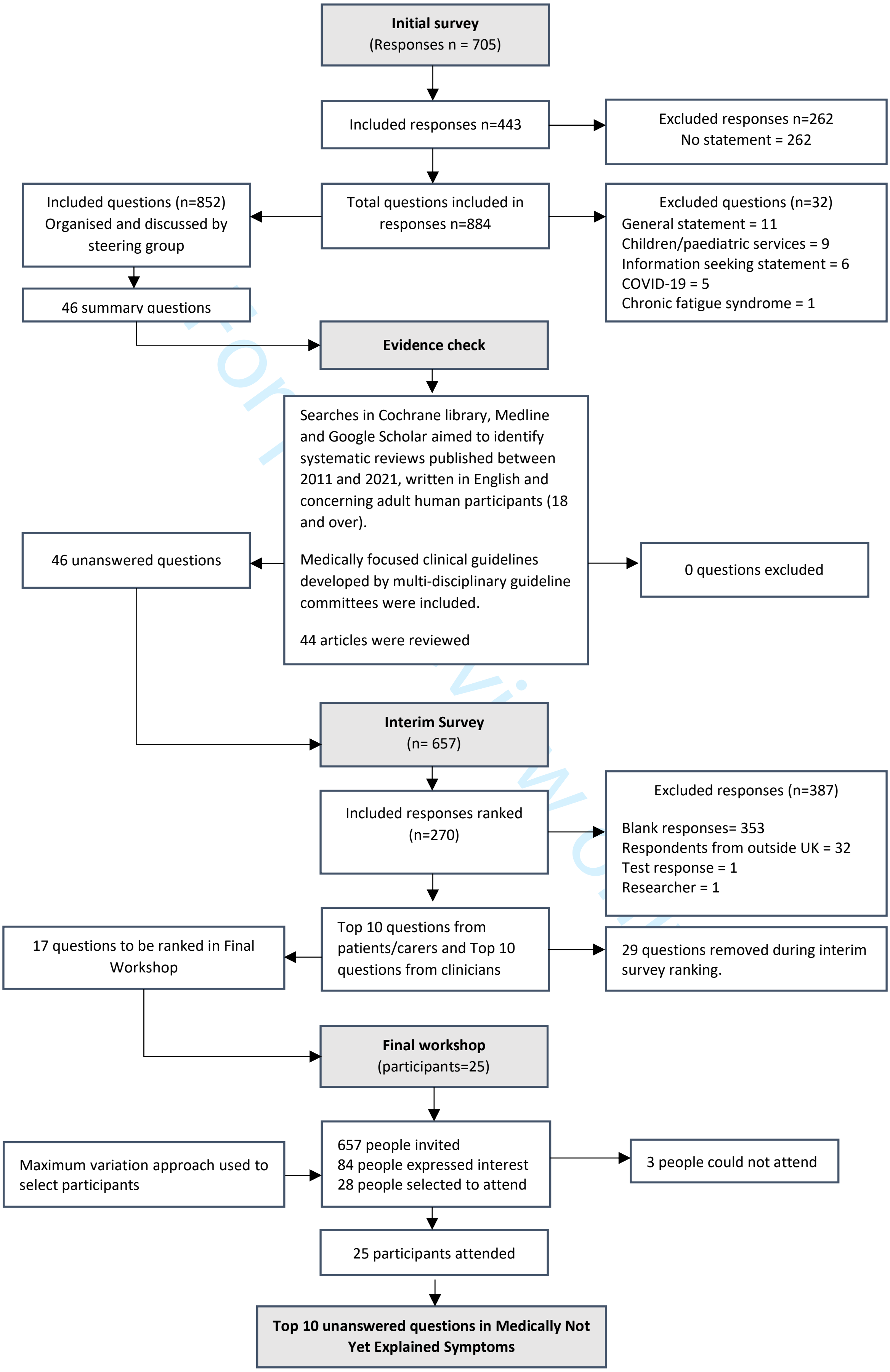

Figure 2: The proportion of questions per domain suggested by patients, caregivers, clinicians and other

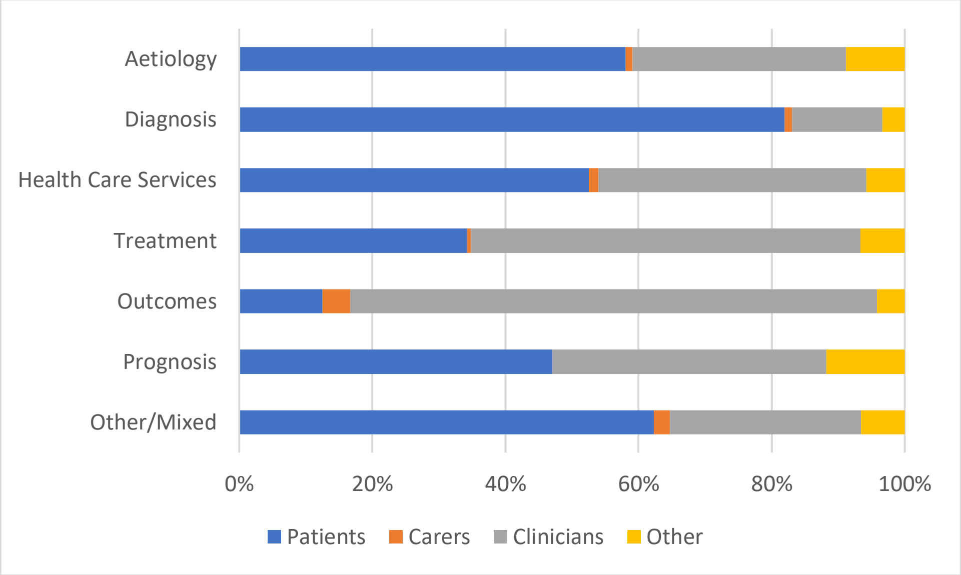

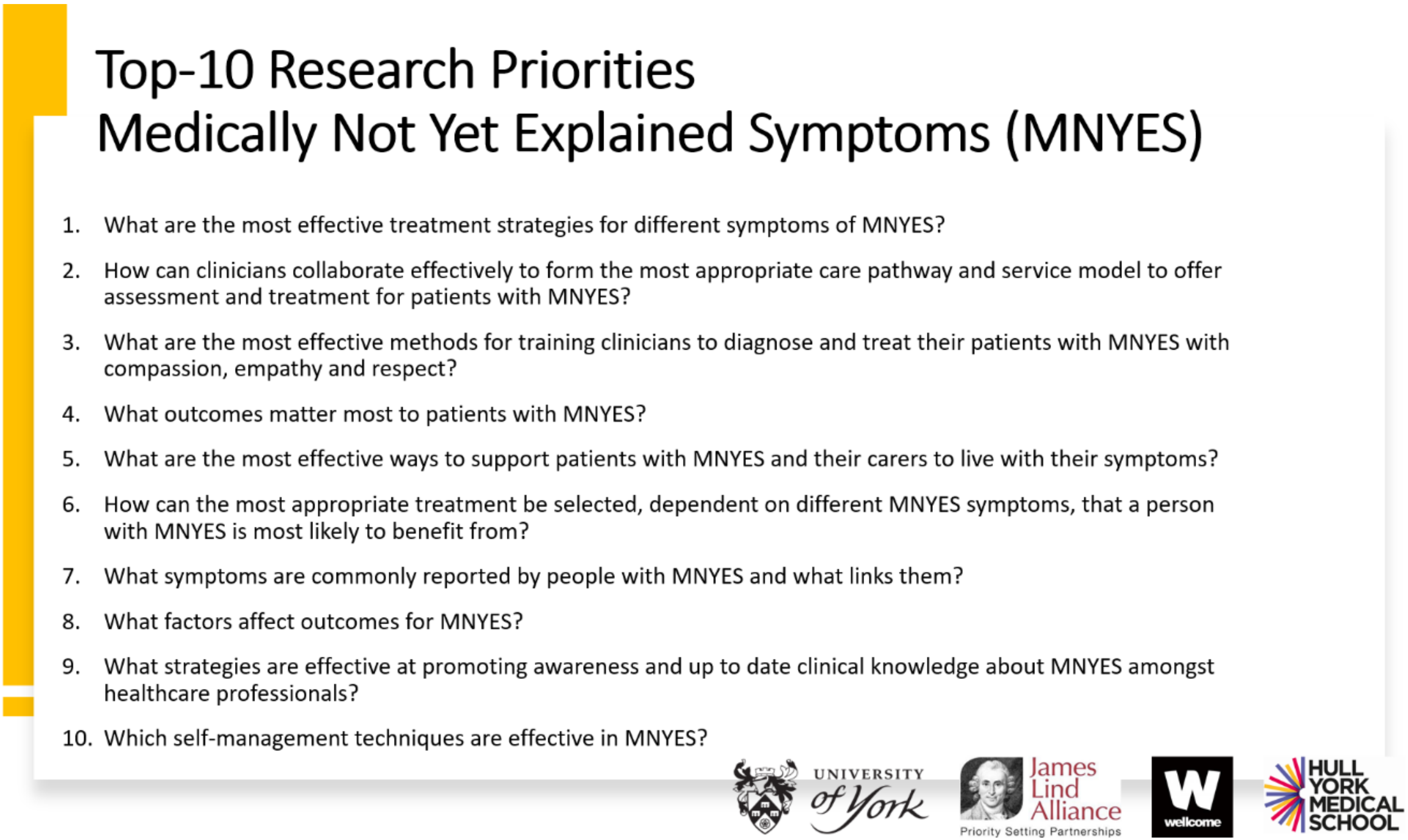

Figure 3: Top 10 Research Priorities for Medically Not Yet Explained Symptoms

For peer review only - http://bmjopen.bmj.com/site/about/guidelines.xhtml

Appendix

Key words included in evidence check searches

Symptom terms	Publication focus
Medically not yet explained symptoms	Guideline
Medically unexplained symptoms (MUS)	Mechanism
Persistent Somatic Symptoms	Pathology
Somatic Symptom and Related Disorders (SSRD)	Development
Chronic pain	Assessment
Unexplained pain	Diagnosis
Fatigue	Investigation
Unexplained fatigue	Treatment
Conversion disorder	Management
Neurological conversion symptoms	Healthcare service
Functional neurological disorder	Patient care
Functional symptoms	Care pathway
Functional neurological symptom	Outcome
Functional cognitive symptom	Prognosis
Functional weakness	
Dissociative disorders	
Dissociative neurological disorder	
Dissociative syncope	
Unexplained dizziness	
Fibromyalgia	
Irritable bowel syndrome (IBS)	
Nonepileptic seizures	

Identifying the top research priorities in medically not yet explained symptoms (MNYES): a James Lind Alliance priority setting partnership

Journal:	*BMJ Open*
Manuscript ID	bmjopen-2022-061263.R1
Article Type:	Original research
Date Submitted by the Author:	18-May-2022
Complete List of Authors:	van der Feltz-Cornelis, Christina; University of York, Dept. of Health Sciences, HYMS; Tees Esk and Wear Valleys NHS Foundation Trust<br>Sweetman, Jennifer; University of York<br>Edwards, Mark; St George's University of London; St George’s University Hospitals NHS Foundation Trust<br>Gall, Nicholas; University of London Kings College Hospital, Department of Cardiology<br>Gilligan, Jennifer; Tees Esk and Wear Valleys NHS Foundation Trust<br>Hayle, Steph; Patient representative<br>Kaul, Arvind; St George’s University Hospitals NHS Foundation Trust<br>Moriarty, Andrew; University of York Department of Health Sciences; Hull York Medical School,<br>Perros, Petros; Royal Victoria Infirmary, Department of Endocrinology<br>Sampford, James; Tees Esk and Wear Valleys NHS Foundation Trust<br>Smith, Natalie; University of York<br>Elfeddali, Iman; Tilburg University, Tranzo Dept.; GGz Breburg, Tilburg, the Netherlands, Centre for Body Mind and Health<br>Varley, Danielle; University of York Department of Health Sciences<br>Gower, Jonathan; JLA
**Primary Subject Heading**:	Evidence based practice
Secondary Subject Heading:	Mental health
Keywords:	Adult psychiatry < PSYCHIATRY, Adult cardiology < CARDIOLOGY, Functional bowel disorders < GASTROENTEROLOGY, Rheumatology < INTERNAL MEDICINE, Adult neurology < NEUROLOGY, PRIMARY CARE

SCHOLARONE™
Manuscripts

I, the Submitting Author has the right to grant and does grant on behalf of all authors of the Work (as defined in the below author licence), an exclusive licence and/or a non-exclusive licence for contributions from authors who are: i) UK Crown employees; ii) where BMJ has agreed a CC-BY licence shall apply, and/or iii) in accordance with the terms applicable for US Federal Government officers or employees acting as part of their official duties; on a worldwide, perpetual, irrevocable, royalty-free basis to BMJ Publishing Group Ltd ("BMJ") its licensees and where the relevant Journal is co-owned by BMJ to the co-owners of the Journal, to publish the Work in this journal and any other BMJ products and to exploit all rights, as set out in our licence.

The Submitting Author accepts and understands that any supply made under these terms is made by BMJ to the Submitting Author unless you are acting as an employee on behalf of your employer or a postgraduate student of an affiliated institution which is paying any applicable article publishing charge ("APC") for Open Access articles. Where the Submitting Author wishes to make the Work available on an Open Access basis (and intends to pay the relevant APC), the terms of reuse of such Open Access shall be governed by a Creative Commons licence – details of these licences and which Creative Commons licence will apply to this Work are set out in our licence referred to above.

Other than as permitted in any relevant BMJ Author's Self Archiving Policies, I confirm this Work has not been accepted for publication elsewhere, is not being considered for publication elsewhere and does not duplicate material already published. I confirm all authors consent to publication of this Work and authorise the granting of this licence.

For peer review only

Identifying the top research priorities in medically not yet explained symptoms (MNYES): a James Lind Alliance priority setting partnership

Christina M van der Feltz-Cornelis,[1,2,3] Jennifer F Sweetman,[1] Mark Edwards,[4,5] Nicholas Gall,[6] Jennifer Gilligan,[3] Steph Hayle,[7] Arvind Kaul,[5] Andrew S Moriarty,[1,2] Petros Perros,[8] James Sampford,[3] Natalie Smith,[1] Iman Elfeddali,[9,10] Danielle Varley,[1] Jonathan Gower[11]

Corresponding author: Christina van der Feltz-Cornelis, Dept. of Health Sciences, HYMS, University of York, ARRC Building, T204, Heslington, York, United Kingdom. Phone +441904321313 Email christina.vanderfeltz-cornelis@york.ac.uk

[1] Dept of Health Sciences, University of York, York, United Kingdom

[2] Hull York Medical School, (HYMS), University of York, York, United Kingdom

[3] Tees Esk and Wear Valley NHS Foundation Trust, Darlington, United Kingdom

[4] Neuroscience Research Centre, Institute of Molecular and Clinical Sciences, St George's University of London, London, United Kingdom.

[5] St. George's University Hospitals NHS Foundation Trust, London, United Kingdom

[6] Department of Cardiology, King's College Hospital, Denmark Hill, London, SE5 9RS, United Kingdom

[7] Patient representative, North Yorkshire, United Kingdom

[8] Department of Endocrinology, Royal Victoria Infirmary, Newcastle upon Tyne, United Kingdom

[9] Tranzo Dept., Tilburg University, Tilburg, the Netherlands

[10] Centre for Body Mind and Health, GGz Breburg, Tilburg, the Netherlands

[11] James Lind Alliance, Southampton, United Kingdom

Abstract

Objectives This study establishes research priorities for Medically Not Yet Explained Symptoms, also known as Persistent Physical Symptoms (PPS) or Medically Unexplained Symptoms (MUS), from the perspective of patients, caregivers and clinicians, in a priority setting partnership (PSP) following the James Lind Alliance (JLA) approach. Research into such symptoms in general has been poorly funded over the years and so far has been primarily researcher-led with minimal input from patients, caregivers and clinicians; and sometimes has been controversial.

Design JLA priority setting partnership method. The PSP termed these symptoms Medically Not Yet Explained Symptoms (MNYES).

Methods The study was conducted according to the JLA's detailed methodology for conducting priority setting exercises. It involved five key stages: defining the appropriate term for the conditions under study by the PSP Steering Group; gathering questions on MNYES from patients, caregivers and clinicians in a publicly accessible survey; checking these research questions against existing evidence; interim prioritisation in a second survey; and a final multi-stakeholder consensus meeting to determine the top 10 unanswered research questions using the modified nominal group methodology.

Results Over 700 responses from UK patients, caregivers and clinicians were identified in the two surveys and charities contributed from a broad range of medical specialties and primary care. The final top 10 unanswered research questions cover, amongst others: treatment strategies, collaborative care pathways, training for clinicians, outcomes that matter to patients, and personalisation of treatment.

Interpretation The top 10 unanswered research questions are expected to generate much needed, relevant and impactful research into MNYES.

Word count: 4000

Key words: James Lind Alliance; Priority Setting; Medically Not Yet Explained Symptoms (MNYES); Medically Unexplained Symptoms (MUS); Persistent Physical Symptoms (PPS)

For peer review only - http://bmjopen.bmj.com/site/about/guidelines.xhtml

Introduction

Medically Not Yet Explained Symptoms (MNYES), also known as Medically Unexplained Symptoms (MUS) or Persistent Physical Symptoms (PPS)(1), represent up to 30% of presentations in primary care and internal medicine settings (2-4). They can include fatigue, pain, dizziness, irritable bowel syndrome, and functional neurological symptoms (FND)(4). They are often deemed to be stress-related, or of psychological origin, and comorbid depressive or anxiety disorder occur in approximately 30% of cases (3). Patients diagnosed with these symptoms often feel that they are not taken seriously, although care may have been taken to explain their condition properly. It can take a long time to reach the conclusion that patients have MNYES; during this time they typically experience high levels of disability and face repeating appointments and diagnostic procedures. They hear that no cause can be found for their symptoms and this is often delivered by clinicians who have a dualistic view of health and disease. Disability and absenteeism occurs frequently even in patients who present only within primary care with a low number of symptoms and where the effect of demographic factores, anxiety and depressive disorder are taken into account(5-8). This inevitably leads to disappointment and frustration (9). Many clinicians lack confidence in the assessment and management of MNYES, or may exhibit behaviours perceived as dismissive. Patients often perceive a stigmatising attitude from clinicians and a sense that they are being judged as neurotic or mentally unwell (10-12). Moreover, management plans may not be sufficiently holistic to address all patient concerns, and effective treatments are scarce. All the above impact negatively on long-term prognosis.

The focus of research on MNYES is often on particular subsets of symptoms, such as chronic pain, chronic fatigue, irritable bowel syndrome, and dizziness, but lacks a comprehensive view. This has ramifications for patients who visit different clinics for their various symptoms, without sustained improvement, and as such experience unmet needs (13, 14). To address this, the University of York through the lead author (CFC) established a Priority Setting Partnership (PSP) for research needed to address MNYES. We engaged with members of the public, patients with MYNES and their caregivers, clinicians of all medical specialties known to have patients with MNYES (15), and other key stakeholders such as charities and the Royal College of Psychiatry Liaison Faculty. Close collaboration with the James Lind Alliance (JLA) enabled this PSP to follow their established, rigorous approach to identify the treatment and

For peer review only - http://bmjopen.bmj.com/site/about/guidelines.xhtml

management priorities of stakeholders (patients, caregivers, clinicians and support organisations) and to incorporate these into a research agenda (16).

The European Association of Psychosomatic Medicine has published a research agenda in this domain with one of the research priorities being patient preferences for research in this field (17). Until now, however, there has been relatively little support available for people with MNYES and those who care for them, to enable them in setting up the research agenda. Engaging patients in the research process incorporates their perspective as ‘experts’ from their unique experience of living with symptoms, as well as their personal knowledge regarding diagnostic trajectories and treatments in the healthcare setting if such symptoms remain (partially) unexplained (18). This study aims to address this knowledge gap.

The aim of this PSP was therefore to develop a research agenda with the joint perspectives of patients, caregivers, clinicians and support organisations across the United Kingdom as the frame of reference, to identify the most important unanswered research questions in MNYES.

Methods

This study was undertaken according to the JLA’s method for undertaking PSPs as delineated in the JLAs Guidebook (16). An independent JLA Adviser (JG) guided the study team through the project and ensured that every step followed the JLA’s methodology and adhered to the JLA’s principles of transparency and balanced inclusion of patients, caregivers and clinicians. All materials related to this PSP can be found on the JLA website (19).

Establishing the Steering Group

In March 2020 the MNYES PSP Steering Group met for the first time. Members of the Steering Group were selected by a snowballing method, asking clinics and clinicians providing diagnosis and treatment of the different conditions potentially covered by MNYES to signpost colleagues with an interest in MNYES. Also, charities, patient networks and PPI networks were approached to identify patients and caregivers. They were invited and selected based upon the capabiliy, motivation and consent to contribute to the JLA PSP working group standards of reference as described in the JLA website. Efforts were made to have a representation of patients with pain, fatigue, FND, IBS and dizziness, as they are the most common MNYES conditions as shown in the literature (20). Efforts were made to include people from areas

For peer review only - http://bmjopen.bmj.com/site/about/guidelines.xhtml

outside of London, including rural areas and underserved areas as delineated by clinics and GPs in the North of England in the Steering Group. The Steering Group was tasked with overseeing the Priority Setting Partnership by having meetings every six weeks, chaired by the JLA advisor, and making critical decisions at key points of the project (19). The composition of the Steering Group is shown in Box 1 (Supplement).

Terminology

Many terms are used for these symptoms, including, but not limited to, Persistent Somatic Symptoms (PPS) (1), Somatic Symptom and Related Disorders (SSRD)(21), Bodily Distress Disorders (BDD) (22), Medically Unexplained Symptoms (MUS), Functional symptoms, and Functional Neurological Disorder (FND). There is an ongoing debate amongst researchers and clinicians about how to refer to these conditions. Many of such terms have been deemed unsatisfactory by patients, caregivers and clinicians as well as researchers for a variety of reasons, leading to ongoing efforts from researchers to find a better term; (23-25) however, so far the patient, carer and clinician perspective regarding the choice of preferred term has been lacking. This may seem semantic, however it underpins the conceptual confusion that exists regarding these symptoms (26). Unfortunately, in some cases such uncertainty can give rise to deeply rooted controversy that ultimately can be traced back to lack of knowledge regarding the underlying conditions, and to related stigma. This knowledge gap could either be a factual lack of evidence, or a lack of availability of existing knowledge to clinicians, patients and the general public alike. Therefore, the study's PSP Steering Group took time to decide what terminology to use in the study.

A common concern appeared to be the distress caused to patients, caregivers and clinicians alike by the lack of adequate explanations, diagnostic methods and treatments for these symptoms – which are often poorly understood across these groups too. This was felt to have a negative impact on clinical work and research pertaining to these conditions and to stigmatise them at a societal level. After deliberation, the PSP Steering Group agreed to use the term *Medically Not Yet Explained Symptoms (MNYES)* to describe the subject matter for the duration of the study. This was an operational definition not intended to add to or replace other definitions already in use, that was constructed to embrace the views of all stakeholders. MNYES was meant to indicate that although some insights might exist, our understanding is still incomplete. This could pertain to biological, psychological and social

For peer review only - http://bmjopen.bmj.com/site/about/guidelines.xhtml

factors, as well as factors involving the trajectory of patients through various healthcare settings. In that sense, the choice of the term MNYES conveys a message of hope, which feeds into the effort to identify research priorities for the condition.

Inclusion and exclusion criteria

The PSP's Steering Group agreed that the remit should include the aetiology, diagnosis and treatment or medical care of patients with MNYES in the UK, as well as the organisation of services, social consequences and long term outcomes including cost implications for patients. Confirmed topics included (but were not limited to): pain, fatigue, dizziness, functional neurological disorder, bowel symptoms, palpitations and syncope. Ages 16 and older were included. Although fatigue as a symptom was considered for inclusion, Chronic Fatigue Syndrome was considered out of scope since there was another PSP addressing this.

Patient and public involvement

A core principle of JLA priority setting partnerships is collaboration between all stakeholders (patients, caregivers, and clinicians) to ensure their views are represented at each stage of the process. At the level of the steering group, patients, caregivers and clinicians were members of the MNYES Steering Group, represented at every meeting, and involved in the development of PSP surveys. They participated in the initial and interim surveys, and were involved in the organisation of uncertainties, the wording of summary questions, and the verification of evidence checking. At the level of the surveys, patients, public and supporting organisations participated in the surveys as shown in Table 1 (Supplement). The final workshop also included patients, caregivers and clinicians in the final prioritisation process to establish the top 10 research priorities for MNYES. Furthermore, there were observers representing supporting organisations and relevant charitable organisations during the final workshop. All Steering Group members were invited to contribute to the dissemination of the surveys; the information shared by this PSP was developed with members from all stakeholder groups. All PSP steering group members were invited to contribute to the article describing the findings and one of them indeed contributed as a co-author.

For peer review only - http://bmjopen.bmj.com/site/about/guidelines.xhtml

Results

The process is shown in the project flowdiagram presented in Figure 1 below.

- Insert **Figure 1. Flow diagram of MNYES question prioritisation processes** –

First survey

The initial survey (June 2020 - January 2021) asked patients, caregivers and healthcare professionals to indicate their priorities for future research related to MNYES (27). There were 705 respondents who accessed the initial survey; 443 provided at least one question or statement. Respondents were 77% female, 86% white. Data from the 2011 census show that 51% of the England and Wales population were female (28) and 86% of the same population were White (29). 68% of the participants were patients or caregivers as reported in Table 2 (Supplement).

The information specialists (DV and JS) and PSP lead (CFC), grouped similar or duplicate questions into 5 domains, generating 96 draft summary questions on aetiology, diagnosis, health care services, treatment, outcomes, prognosis, and other. Those 96 draft questions were reviewed by small groups of PSP Steering Group members that comprised clinicians, patients and caregivers. Further consolidations were made resulting in 46 summary questions which were reviewed again and signed off at a meeting of the whole PSP Steering Group. A document illustrating this is available on the JLA website (30). Of these 46 questions, 22% related to aetiology, 24% to health and clinical services, 15% to diagnosis, 24% to the treatment of MNYES, and 15% to outcomes. The proportion of questions posed by stakeholder groups, organised by topic, is shown in Figure S1 (Supplement).

Evidence check

The 46 summary questions were checked against published systematic reviews and clinical guidelines. We found that none of the 46 summary questions had been fully answered by previous research; some questions had been answered for specific symptoms, but not comprehensively across all MNYES symptoms. At a subsequent meeting, the Steering Group reviewed the 46 summary questions in relation to the original questions and statements from

For peer review only - http://bmjopen.bmj.com/site/about/guidelines.xhtml

which they derived. This resulted in minor changes to the wording of these 46 questions which were then included in the interim prioritisation survey.

Interim survey

This online survey was completed by 270 participants from across the UK. Patients and caregivers made up 74% of the participants. Demographic information is shown in Table 2 (Supplement).

Final priority setting workshop

The final priority setting workshop was conducted remotely over two days. In total, 25 people participated in the workshop sessions; four JLA advisors facilitated the subgroups, eight people observed and one person provided technical support. Participants included 11 people with MNYES or caregivers, and 14 healthcare professionals representing psychiatry, general practice, stroke, neurology, physiotherapy, psychology, occupational therapy and gastroenterology. The final top 10 research priorities were agreed by consensus between all the participants as listed in Figure 2. They were placed on the James Lind Alliance Website (31).

- Insert **Figure 2. Top 10 research priorities** -

The research priorities which were ranked 11 – 17 are also listed on the JLA website (31) and in Box 2 (Supplement).

Discussion

In this study, we used the JLA Priority Setting Partnership processes to identify the top 10 unanswered research questions for MNYES. We utilised the collective perspectives of patients, caregivers, and clinicians, and focused on areas where up-to-date, reliable research evidence is lacking. This process was supported by charitable and professional organisations across the United Kingdom. The study highlighted the paucity of evidence-driven practice in MNYES care since none of the 46 research questions gathered from our survey have been previously answered by level I evidence. Based on the extensive discussions that took place during the

For peer review only - http://bmjopen.bmj.com/site/about/guidelines.xhtml

meetings, the following major themes emerged from the top 10 unanswered research questions.

Theme 1: Treatment

The first and sixth research priorities concern treatment. The first, *What are the most effective treatment strategies for different symptoms of MNYES?* pertains to all potential treatment strategies (for example: pharmacological, psychological, physical, or collaboration models) to help manage or alleviate any MNYES or combination of symptoms, such as pain, fatigue, dizziness, functional neurological disorder, bowel symptoms, palpitations and syncope. The sixth research priority, *How can the most appropriate treatment be selected, dependent on different MNYES symptoms, that a person with MNYES is most likely to benefit from?* focuses on the best ways to match people who experience specific MNYES with the treatments that are most likely to benefit them. This relates to personalisation of treatment based on diagnostic factors, such as a history of trauma, biomarkers or treatment needs. Given the high unmet clinical need and the heterogenity of MNYES, it is no surprise that this is considered to be an important research priority. Interestingly, this was also a research priority identified by the Delphi study conducted amongst experts in the field on behalf of the European Association of Psychosomatic Medicine (17); treatment and personalised treatment are therefore considered to be research priorities which are widely supported in this field.

Theme 2: The Role of Clinicians

Several research priorities involve the role of the clinicians in the diagnostic and treatment process. The second research priority, *How can clinicians collaborate effectively to form the most appropriate care pathway and service model to offer assessment and treatment for patients with MNYES?* pertains to the role that clinicians play towards each other. It includes the professionals and services which may be involved for the purpose of assessment, diagnosis and the provision or coordination of treatment. It relates to the fact that often many different clinicians provide diagnostic assessements to people with MNYES, or are sought to provide treatment to them. This priority is focused on finding the best ways for clinicians to collaborate with each other to form an appropriate care pathway to support people with MNYES. This could be psychiatric consultation models, multi-disciplinary team models, collaborative care

For peer review only - http://bmjopen.bmj.com/site/about/guidelines.xhtml

models or other integrated care pathways. The third research priority, *What are the most effective methods for training clinicians to diagnose and treat their patients with MNYES with compassion, empathy and respect?* refers to medical practitioners who are already qualified and employed in positions that involve the diagnosis and treatment of people with MNYES. The focus of attention here is on the communication of information which acknowledges the perspective and concerns of the person experiencing MNYES. Priority five, *What are the most effective ways to support patients with MNYES and their carers to live with their symptoms?* focuses on identifying the range of different options for supporting people with MNYES and their caregivers. These may include but are not limited to: finding the most appropriate and acceptable ways to discuss MNYES with people who experience them and their caregivers; guidance about how to discuss MNYES with family members and friends; shared decision making when discussing treatment options; peer support organisations; reasonable adjustments in employment settings; adaptations to accommodation; psychological support to cope while living with symptoms; recovery and rehabilitation approaches. Another research priority fitting with this theme is priority nine: *What strategies are effective at promoting awareness and up to date clinical knowledge about MNYES amongst healthcare professionals?* This priority emphasises strategies to consistently and effectively ensure that clinicians are aware of and have the most up to date information about MNYES which will enable the care people receive to reflect current evidence. Given the existing knowledge gaps this is considered an important priority.

Theme 3: Symptoms and Outcomes

Patients were involved in all discussions regarding research priorities. However, some research priorities mention the patient perspective explicitly, such as, for example, priority four, *What outcomes matter most to patients with MNYES?* Based upon the survey answers, these may include, but are not limited to: symptom reduction; changes in biomarkers; improvements in abilities to undertake daily tasks; improvements in quality of life; individual goal achievements; or improvements in functioning. Priority seven, *What symptoms are commonly reported by people with MNYES and what links them?* relates to the fact that the list of MNYES is extensive and people who experience these symptoms often report living with multiple MNYES. The aim of this priority is to identify the symptoms which most commonly co-occur and the underlying factors and mechanisms between them. For priority eight, *What*

For peer review only - http://bmjopen.bmj.com/site/about/guidelines.xhtml

factors affect outcomes for MNYES? it was agreed the diversity of factors affecting outcomes should include biomarkers, psychological factors and trauma, health services-related issues such as feedback from recovered patients about what helped them, the availability and quality of information clinicians have access to, the way information is shared between clinicians and people experiencing MNYES, and social factors such as poverty, education, family dysfunction or domestic abuse, and work environment. Given the number of questions that pertained to aetiological factors, and the fact that the related uncertainty plays a role in the choice of MNYES as a term, this can be considered an important research theme.

Theme 4: Recovery

Research priority number ten relates to recovery: *Which self-management techniques are effective in MNYES?* this priority concerns the strategies that people experiencing MNYES can use separately from visits to clinics. The focus is to identify the most effective self-administered therapies for managing or alleviating MNYES, used separately, or in combination with formal treatment. Examples of self-management approaches include education, exercise, and dietary changes. It should be noted that this research priority, in contrast to ones covered by the other themes, considers that recovery in MNYES can occur, either by recovery of symptoms, or by personal recovery with ongoing symptomatology. Recovery of symptomatology is referred to as clinical recovery and is covered by the other themes. Recovery whilst symptoms are ongoing is called personal recovery (32), meaning that despite symptoms being present, function has to some extent been restored through treatment, self-management or disability management.

In mental health research and clinical practice, especially in relation to psychotic conditions, personal recovery is a construct that has increasingly gained attention over the past thirty years; however, so far the term has not been used in the context of MNYES. Generally, the emphasis, both in clinical practice and in research, seems to have been to either attempt to attain clinical recovery, or to send the patient home with the message that MNYES cannot be cured and that one would have to live with the condition. This dichotomy has fed into the ongoing controversy about how to approach MNYES. This polarising stance is unhelpful. Developing this research priority would require embracing the concept that personal recovery refers to an individual process of adaptation and development where one does not simply

For peer review only - http://bmjopen.bmj.com/site/about/guidelines.xhtml

return to, but rather grows beyond the premorbid self (33), with an emphasis on the patient perspective. It could provide an important contribution to the further development of research in this domain, alongside the research priorities summarised in the other themes.

Strengths of the study

This is the first study establishing research priorities for MNYES, according to the JLA Priority Setting Partnership method which incorporates the views of patients, caregivers and clinicians. The use of established and transparent James Lind Alliance methodology is a strength of this study.

This is a highly contentious area; however, the research priorities were widely supported by over 400 participants including clinicians from a variety of disciplines, patients with a range of symptoms, caregivers, charitible organisations, and other supporting organisations Over 700 responses were gathered from patients, caregivers and clinicians from an array of medical specialties including primary care, indicating that the priorities were widely supported. Contributions of people from ethnic and gender minority groups and from underserved areas within the PSP Steering Group, surveys and final workshop supports the inclusive nature of this work and indicates these priorities are important to a diverse range of people.

The themes identified in this PSP cover a broad range of ideas, issues and uncertainties; these are outlined in the top 10 research priorities and also reflected in the seven research priorities that did not make the top 10. Research priorities 11, 12 and 17 would link well with theme 3 in exploring associations of MNYES with mental health and somatic comorbidity, as well as the development of symptoms over time. Priorities 13 and 14 would fit in theme two, the role of clinicians; 15 and 16 link with theme one, treatment. This suggests that the themes covered by the top 10 priorities are consistent with the other research priorities which were proposed during this priority setting process.

Limitations of the study

When comparing the participants of survey 1 with survey 2, there were 443 participants in survey 1, and 270 in survey 2. The final workshop was attended by 25 people. These are high

For peer review only - http://bmjopen.bmj.com/site/about/guidelines.xhtml

numbers and certainly adequate for priority setting according to the JLA method. However, as the description of the roles is self-described, the variety of investigative participants remains unclear in some respects. For example, it should be pointed out that in Table 2, 10 people self described as "doctor," and 8 as "other" clinician and they may well have been doctors working in primary care as GPs, or rheumatologists; however, we do not know for sure. Regarding the patients, they would state their self-described main symptom as "pain" in approximately half of the cases; from their answers to the open questions, it emerged that this often would refer to musculo-skeletal or rheumatological pain. So, while the exact variety is uncertain, however, it is unlikely that this contributed to priorities in the final list of issues related to MNYES.

The study provides a good overview of research priorities for MYNES in the UK, however given the specific cultural aspects and health care organisation in the UK, the findings may not be generalisable to other countries. A similar PSP is currently being conducted in the Netherlands and may shed light on research priorities in a non-NHS healthcare setting. This will provide an opportunity to compare and evaluate the generalisability of these findings and the influence of different cultural and healthcare settings. Future research highlighting the situation in Low and Middle Income Countries would be beneficial. The results of this PSP will enable funders to prioritise research in MNYES as outlined here and hopefully will provide new, much needed knowledge in this domain.

Conclusion

MYNES are common and reflect a high level of unmet clinical need. Incorporating patient-driven research in MNYES research can allow researchers to better address the complex care needs of people with MNYES. The most important aspect of this priority setting exercise was strengthening the relationship between patients, caregivers, clinicians and support organisations and generating a list of priorities valued by these stakeholders, which we hope will guide future research.

We have identified the top 10 research priorities in MNYES using the rigorous JLA priority setting method that may open the door to further research addressing the knowledge gaps and controversies in this area, and hopefully alleviate some of the stigma related to these conditions and the people suffering from MNYES. Identification of these top 10 research

For peer review only - http://bmjopen.bmj.com/site/about/guidelines.xhtml

priorities is an important first step to generating relevant, impactful research that will ultimately improve the lives of people with MNYES.

Acknowledgements: The Steering Group would like to thank to all the patients, caregivers, families, friends, healthcare professionals and supporting organisations who contributed to this work. In addition to the co-authors, Philippa Bolton, Sally Brabyn, Tracey Cunningham, Rosie Evans, Miriam Lomas, Margot Metz, Chris Price, Annie Shaw, Scott Spain, Lesley Spain were members of the PSP Steering Group. The following people who attended the final workshop for priority setting agreed to be named: Anna Burneika, Kit Byatt, Phoebe Cole, Tracey Cunningham, Mark Edwards, Rosie Evans, Eve Fazakerley, Jennifer Gilligan, Stephanie Johnston, Claire Jones, Hilary Lewis, Joseph Littlewood, Miriam Lomas, Andrew Moriarty, Elizabeth Paul, Emma Reinhold, Keziah Reiss, James Sampford, Annie Shaw, Gemma Smith, Martin Veysey, Juliet Wilson, Jennifer Wilson.

Ethics statement: Ethical approval was not required as confirmed by the research governance board of the Department of Health Sciences of the University of York.

Data availability statement

Data relevant to the study are included in the article or on the James Lind Alliance website (https://www.jla.nihr.ac.uk/priority-setting-partnerships/medically-not-yet-explained-symptoms/) and linked University of York website (https://www.york.ac.uk/healthsciences/research/mental-health/projects/mnyes/).

Conflicts of interest statement

The authors have no conflicts of interest to declare.

Author statement

CFC was the project lead, instigating the application to the JLA. CFC, NS, JFS and JG organised the Steering Group meetings. JFS and DV designed and built the surveys, analysed the data, and conducted the evidence check under supervision of JG and CFC. CFC, JFS, and NS wrote the majority of the final manuscript. JG (JLA Chair) chaired all the meetings, led the consensus workshop and ensured compliance with methodology throughout. NS and JFS took minutes for Steering Group meetings, built and distributed surveys, and organised the consensus

workshop. JFS conducted the searches for the evidence check and screened the results with CFC. Members of the Steering Group CFC, JFS, ME, NG, JG, SH, AK, ASM, PP, JS, NS, IE, DV, PB, SB, TC, RE, ML, MM, CP, AS, SS, LS all attended a majority of the meetings, agreed the initial protocol and the evidence check protocol, piloted and signed off the surveys and disseminated them, checked the raw questions against the indicative ones, reviewed the evidence check results and agreed the final longlist. TC, ME, JG, ML, ASM, JFS, and AS were present at the final consensus workshop. All authors reviewed and contributed to the final manuscript and approved it prior to submission.

Funding

This work was part-funded by the Wellcome Trust [ref: 204829] from the overall Wellcome Trust ISSF award through the Centre for Future Health (CFH) at the University of York.

For peer review only

For peer review only - http://bmjopen.bmj.com/site/about/guidelines.xhtml

References

1. Patel M, James K, Moss-Morris R, Husain M, Ashworth M, Frank P, Ferreira N, Mosweu I, McCrone P, Hotopf M, David A, Landau S, Chalder T. Persistent physical symptoms reduction intervention: a system change and evaluation (PRINCE)-integrated GP care for persistent physical symptoms: protocol for a feasibility and cluster randomised waiting list, controlled trial. BMJ Open. 2019 Jul 23;9(7):e025513. doi: 10.1136/bmjopen-2018-025513.
2. Hoedeman R, Blankenstein AH, van der Feltz-Cornelis CM, Krol B, Stewart R, Groothoff JW. Consultation letters for medically unexplained physical symptoms in primary care. Cochrane Database of Systematic Reviews. 2010(12).
3. Van Hemert AM, Hengeveld MW, Bolk JH, Rooijmans HG, Vandenbroucke JP. Psychiatric disorders in relation to medical illness among patients of a general medical out-patient clinic. Psychological medicine. 1993;23(1):167-73.
4. Kirmayer LJ, Groleau D, Looper KJ, Dao MD. Explaining medically unexplained symptoms. The Canadian journal of psychiatry. 2004;49(10):663-72.
5. van der Leeuw G, Gerrits MJ, Terluin B, Numans ME, van der Feltz-Cornelis CM, van der Horst HE, Penninx BW, van Marwijk HW. The association between somatization and disability in primary care patients. J Psychosom Res. 2015 Aug;79(2):117-22. doi: 10.1016/j.jpsychores.2015.03.001. Epub 2015 Mar 11. PMID: 25824596.
6. den Boeft M, Twisk JW, Hoekstra T, Terluin B, Penninx BW, van der Wouden JC, Numans ME, van der Horst HE. Medically unexplained physical symptoms and work functioning over 2 years: their association and the influence of depressive and anxiety disorders and job characteristics. BMC Fam Pract. 2016 Apr 14;17:46. doi: 10.1186/s12875-016-0443-x. PMID: 27079909; PMCID: PMC4831095.
7. Hoedeman R, Krol B, Blankenstein N, Koopmans PC, Groothoff JW. Severe MUPS in a sick-listed population: a cross-sectional study on prevalence, recognition, psychiatric co-morbidity and impairment. BMC Public Health. 2009 Dec 1;9:440. doi: 10.1186/1471-2458-9-440. PMID: 19951415; PMCID: PMC2793259.
8. Escobar JI, Waitzkin H, Silver RC, Gara M, Holman A. Abridged somatization: a study in primary care. Psychosom Med. 1998 Jul-Aug;60(4):466-72. doi: 10.1097/00006842-199807000-00012. PMID: 9710292.
9. Salmon P, Peters S, Stanley I. Patients' perceptions of medical explanations for somatisation disorders: qualitative analysis. BmJ. 1999;318(7180):372-6.
10. Tolchin B, Tolchin DW, Stein MA. How Should Clinicians Minimize Harms and Maximize Benefits When Diagnosing and Treating Disorders Without Biomarkers? AMA journal of ethics. 2021;23(7):530-6.
11. Hatcher S, Arroll B. Assessment and management of medically unexplained symptoms. Bmj. 2008;336(7653):1124-8.
12. Stone J, Wojcik W, Durrance D, Carson A, Lewis S, MacKenzie L, et al. What should we say to patients with symptoms unexplained by disease? The “number needed to offend”. Bmj. 2002;325(7378):1449-50.
13. Carson AJ, Ringbauer B, Stone J, McKenzie L, Warlow C, Sharpe M. Do medically unexplained symptoms matter? A prospective cohort study of 300 new referrals to neurology outpatient clinics. Journal of Neurology, Neurosurgery & Psychiatry. 2000;68(2):207-10.
14. Reid S, Wessely S, Crayford T, Hotopf M. Frequent attenders with medically unexplained symptoms: service use and costs in secondary care. The British Journal of Psychiatry. 2002;180(3):248-53.
15. Nimnuan C, Hotopf M, Wessely S. Medically unexplained symptoms: an epidemiological study in seven specialities. Journal of psychosomatic research. 2001;51(1):361-7.
16. James Lind Alliance. The James Lind Alliance Guidebook Version 7. 2018.
17. van der Feltz-Cornelis CM, Elfeddali I, Werneke U, Malt UF, Van den Bergh O, Schaefert R, et al. A European research agenda for somatic symptom disorders, bodily distress disorders, and

For peer review only - http://bmjopen.bmj.com/site/about/guidelines.xhtml

functional disorders: results of an estimate-talk-estimate Delphi expert study. Frontiers in psychiatry. 2018;9:151.
18. Gill PJ, Cartwright E. Partnering with patients in the production of evidence. Royal Society of Medicine; 2021.
19. James Lind Alliance. Priority Setting Partnership: Medically Not Yet Explained Symptoms 2021 [Available from: https://www.jla.nihr.ac.uk/priority-setting-partnerships/medically-not-yet-explained-symptoms/.
20. Kellner R & Sheffield BF. The one-week prevalence of symptoms in neurotic patients and normals. Am J Psychiatry 1973;50:734-41
21. American Psychiatric Association. Diagnostic and statistical manual of mental disorders: DSM-5. Arlington, VA. 2013.
22. World Health Organization. International Classification of Diseases-11 (ICD-11)2019.
23. van der Feltz-Cornelis CM, Elfeddali I, Werneke U, Malt UF, Omer Van den Bergh O, Schaefert R, Kop WJ, Lobo A, Sharpe M, Söllner W, Löwe B. A European Research Agenda for Somatic Symptom Disorders, Bodily Distress Disorders, and Functional Disorders: Results of an Estimate-Talk-Estimate Delphi Expert Study. Front Psychiarty 2018 May 14;9:151. DOI: 10.3389/fpsyt.2018.00151
24. Ding JM, Kanaan RA. What should we say to patients with unexplained neurological symptoms? How explanation affects offence. J Psychosom Res. 2016 Dec;91:55-60. doi: 10.1016/j.jpsychores.2016.10.012.
25. Stone J, Wojcik W, Durrance D, Carson A, Lewis S, MacKenzie L, Warlow CP, Sharpe M. What should we say to patients with symptoms unexplained by disease? The "number needed to offend". BMJ. 2002 Dec 21;325(7378):1449-50.
26. Van der Feltz-Cornelis C, Van Dyck R. The notion of somatization: an artefact of the conceptualization of body and mind. Psychotherapy and psychosomatics. 1997;66(3):117-27.
27. University of York. Research priority setting for Medically Not Yet Explained Symptoms (MNYES) in an Anglo-Dutch partnership with the James Lind Alliance 2021 [Available from: https://www.york.ac.uk/healthsciences/research/mental-health/projects/mnyes/.
28. GOV.UK. Male and female populations 2018 [Available from: https://www.ethnicity-facts-figures.service.gov.uk/uk-population-by-ethnicity/demographics/male-and-female-populations/latest.
29. GOV.UK. Population of England and Wales 2018 [Available from: https://www.ethnicity-facts-figures.service.gov.uk/uk-population-by-ethnicity/national-and-regional-populations/population-of-england-and-wales/latest.
30. https://www.jla.nihr.ac.uk/priority-setting-partnerships/medically-not-yet-explained-symptoms/downloads/medically-not-yet-explained-symptoms-psp-all-questions-data.doc
31. James Lind Alliance. Medically Not Yet Explained Symptoms Top 10 priorities 2021 [Available from: https://www.jla.nihr.ac.uk/priority-setting-partnerships/medically-not-yet-explained-symptoms/top-10-priorities.htm.
32. Slade M, Amering M, Oades L. Recovery: an international perspective. Epidemiology and Psychiatric Sciences. 2008;17(2):128-37.
33. Slade M. Personal recovery and mental illness: A guide for mental health professionals: Cambridge University Press; 2009.

For peer review only

Figure 1: Flow diagram of MNYES question prioritisation processes

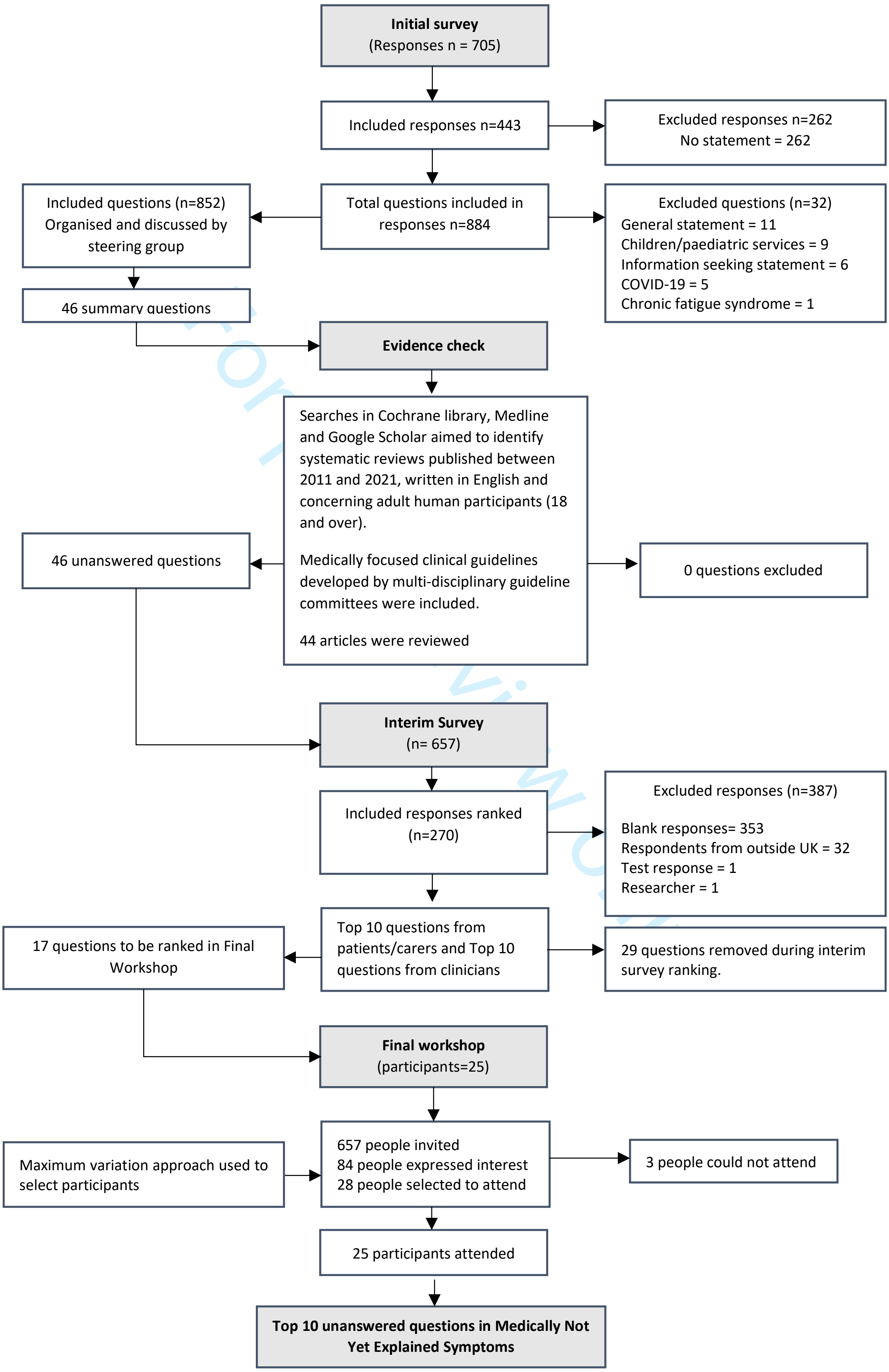

Top-10 Research Priorities
Medically Not Yet Explained Symptoms (MNYES)

1. What are the most effective treatment strategies for different symptoms of MNYES?
2. How can clinicians collaborate effectively to form the most appropriate care pathway and service model to offer assessment and treatment for patients with MNYES?
3. What are the most effective methods for training clinicians to diagnose and treat their patients with MNYES with compassion, empathy and respect?
4. What outcomes matter most to patients with MNYES?
5. What are the most effective ways to support patients with MNYES and their carers to live with their symptoms?
6. How can the most appropriate treatment be selected, dependent on different MNYES symptoms, that a person with MNYES is most likely to benefit from?
7. What symptoms are commonly reported by people with MNYES and what links them?
8. What factors affect outcomes for MNYES?
9. What strategies are effective at promoting awareness and up to date clinical knowledge about MNYES amongst healthcare professionals?
10. Which self-management techniques are effective in MNYES?

UNIVERSITY of York | James Lind Alliance Priority Setting Partnerships | wellcome | HULL YORK MEDICAL SCHOOL

Figure 2: Top 10 Research Priorities for Medically Not Yet Explained Symptoms

Appendix

Key words included in evidence check searches

**Symptom terms**	**Publication focus**
Medically not yet explained symptoms	Guideline
Medically unexplained symptoms (MUS)	Mechanism
Persistent Somatic Symptoms	Pathology
Somatic Symptom and Related Disorders (SSRD)	Development
Chronic pain	Assessment
Unexplained pain	Diagnosis
Fatigue	Investigation
Unexplained fatigue	Treatment
Conversion disorder	Management
Neurological conversion symptoms	Healthcare service
Functional neurological disorder	Patient care
Functional symptoms	Care pathway
Functional neurological symptom	Outcome
Functional cognitive symptom	Prognosis
Functional weakness	
Dissociative disorders	
Dissociative neurological disorder	
Dissociative syncope	
Unexplained dizziness	
Fibromyalgia	
Irritable bowel syndrome (IBS)	
Nonepileptic seizures	

For peer review only - http://bmjopen.bmj.com/site/about/guidelines.xhtml

Acquiring funding, international aspect, and establishing support by the James Lind Alliance

Funding for the study was acquired by the PSP lead (CFC) in November 2019. Part of the collaboration was envisioned to support setting up a parallel PSP Steering Group to explore the same question in the Netherlands, in order to have an international perspective. This article focuses on the description of the process in the UK. Support by the James Lind Alliance Priority Setting Partnership was secured in December 2019 and a JLA Advisor (JG) appointed to the project.

Box 1: Steering group composition

Clinicians: One general practitioner(GP); consultants in cardiology (1), neurology (1), endocrinology (1), clinical liaison psychiatry (3) and rheumatology (1) one stroke physician, a clinical psychologist and one physiotherapist.

Patients and caregivers: Four patients with a variety of pain, fatigue and functional neurological symptoms; one caregiver;

Other: Two information specialists and two project coordinators to facilitate the PSP working group. They prepared meeting documents, surveys, supported recruitment, completed evidence checking and analysis but did not engage in the priority setting.

The consultative process

The study followed several steps in order to establish research priorities, using a mix of online surveys and a priority setting workshop. We created a website to host the surveys (27) and sent out the website link for our partner organisations to distribute within their networks.

Gathering uncertainties

The initial survey was launched between June 2020 and January 2021 and asked patients, caregivers and healthcare professionals to indicate their priorities for future research related to MNYES (27). We promoted the survey through partner organisations' websites, and their social media platforms. In addition, the clinics of clinicians involved in the PSP Steering Group distributed the QR code to their patients. We sent out email reminders to members of professional associations, sent Twitter invitations and placed the survey on the University of York and JLA websites. Distribution of the survey was supported by the members of the PSP, several associations, networks, networking groups and charities. An overview of supporting

For peer review only

organisations is shown in Table 1, below. Some of them collaborated because they found that patients with unexplained symptoms often visit their websites and related specialists to assess their symptoms.

Table 1: Supporting organisations who have promoted this work

Charities	Professional networks	Other networks and Social media groups
British Thyroid Foundation	Allied Health Professional FND Networking groups	INCLUDE (University of York Disability Network)
Fibromyalgia Association UK	Clinical special interest groups	James Lind Alliance
FND Hope	Liaison psychiatry networks	People who have shared information with their friends and family
Graham Hughes International	Royal College of Psychiatrists	Individuals and organisations active on twitter
Guts UK		Special interest and support groups on facebook
Health Unlocked		
Pain UK		
POTS-UK		

We targeted patients with MNYES such as pain, bowel problems, functional neurlogical symptoms, or with diagnoses which comprise a set of symptoms which are medically unexplained such as postural orthostatic tachycardia syndrome (PoTS); their caregivers; and the clinicians who treat them, to participate. Survey respondents were asked whether they had experienced MYNES, in which case they were to select their symptoms from a list, or were healthcare professionals, in which case they were asked to state their profession. Anonymity was preserved in all cases accordance with General Data Protection regulations.

Data analysis and verifying uncertainties

We downloaded the online survey results through Qualtrics and exported them into Microsoft Excel. The responses to this survey were organised into summary questions and these were then checked to make sure that they had not already been answered by research.(16) Our health information specialists (DV and JS) and PSP lead (CFC) grouped the responses into themes and each was analysed in small groups by 3 or 4 members of the Steering Group; one member of each small group was a patient or caregiver and the other members were healthcare professionals. Summary questions were developed, which encapsulated groupings of similar responses to the survey. Responses were removed if they were outside the scope

For peer review only

of the survey or general statements which would be unanswerable by research. The outputs from the small group discussions were reviewed at a subsequent meeting of the full PSP Steering Group.

One health information specialist (JS) checked each in-scope question with existing clinical guidelines published by multi-disciplinary guideline committees and systematic reviews, to ensure that the question had not been already fully answered with high-level evidence. 44 articles were shortlisted from searches conducted in the Cochrane Library, Medline and Google Scholar; key words for the search used are included in the appendix. Reference lists of these studies were also reviewed to identify further relevant references. Articles published between 2011 and 2021 were considered relevant by the Steering Group. Each question was coded to indicate whether it was 'answered'; 'partially answered' or 'unanswered' through the research literature.

Interim priority setting

Between May and July 2021, the second online survey asked participants to select their top 10 priorities from the list of uncertainties presented (19). The second survey was launched independently from the first survey. Questions were presented in a random order to each survey participant to reduce bias. The priorities of patients and caregivers and the priorities from healthcare professionals were ranked separately. The top 10 priorities identified by patients and caregivers were then combined with the top 10 priorities from healthcare professionals to create a shortlist of 17 priorities as there were 3 overlapping priorities.

Final workshop

The 17 top ranked questions were taken forward to the final priority setting consensus meeting that took place virtually on 16th and 17th September 2021. We invited volunteers from supporting organisations, two of whom joined the meeting. We also invited individuals who took part in the online survey to participate in the meeting. 25 participants took part in the workshop comprising 10 patients with a variety of MNYES symptoms, 1 caregiver, and 14 health care professionals. The workshop participants were divided into four groups, each with a balanced mix of patients/caregivers and clinicians. Each group also had observers who did not participate in the process, to fulfill the requirement of transparency of the JLA process. Each group was asked to rank the uncertainties through group discussions using the modified

For peer review only

nominal group technique facilitated by an independent JLA Advisor. Each participant was provided with a printed and electronic copy of the different 'unanswered' questions, and the JLA Advisor leading each group shared an image containing the unanswered questions to aid the group in ranking the 17 uncertainties. The rankings from the four groups were combined and all the workshop participants then came together to discuss these rankings. Similarities and differences between the group rankings were highlighted by the JLA facilitator leading the workshop (JG) and participants were invited to comment on the initial combined ranking. Following this, participants were reallocated into four new groups, maintaining the representation of patients/caregivers and clinicians within each group, to consider the combined group ranking from the previous session. During this session, there was a specific focus placed on the ordering of uncertainties across the top 17 unanswered questions. The rankings from each of the four groups were once again combined and were presented to the whole group for discussion. By the end of the priority setting meeting, we reached consensus on the top 10 UK research priorities for MNYES.

The respondents were 77% female, 86% white; data from the 2011 census show that 51% of the England and Wales population were female (28) and 86% of the same population were White (29). Of the 884 statements, 852 were in scope and 32 out-of-scope. The out-of-scope submissions included general statements (N = 11), children/paediatric services (N = 9), information seeking statements (N = 6), or pertaining to COVID-19 (N = 5) and chronic fatigue syndrome (N = 1).

Table 2: Participant demographic information

	**Initial Survey** (n=443)	**Interim Survey** (n=270)	**Final Workshop** (n=25)***
**Gender (%)**			
Male	89 (20)	33 (12)	6 (24)
Female	339 (77)	227 (84)	18 (72)
Other	14*(3)	2** (1)	1 (4)
**Ethnicity (%)**			
Asian/ Asian British	12 (3)	5 (2)	-
Arab	1 (<1)	-	-
Black/ Black British	3 (1)	2 (1)	-
Mixed/ Multiple	7 (2)	7 (2)	-
White	381 (86)	242 (90)	25 (100)
Other	17 (4)	4 (1)	-
**Age**, mean (SD)	47.65 (12.15)	42.55 (13.26)	39.29 (13.74)
**Experience (%)**			
Patient	288 (65)	183 (68)	10 (40)

For peer review only

Caregiver	13 (3)	17 (6)	1 (4)
Clinician	121 (27)	65 (24)	14 (56)
Other	21 (5)	5 (2)	-
**MNYES symptoms as self-described(%)**			
Pain	219 (49)	147 (54)	6 (24)
Fatigue	236 (53)	146 (54)	8 (32)
Dizziness	156 (35)	91 (34)	5 (20)
Heart palpitations/fainting	134 (30)	83 (31)	5 (20)
Stomach/bowel problems	197 (44)	119 (44)	5 (20)
Problems with movement (tremors, shakes, weakness)	133 (30)	114 (42)	6 (24)
Unexplained blackouts, seizures, sudden loss of awareness	54 (12)	60 (22)	3 (12)
Other (e.g. brain fog, eye and skin problems)	126 (28)	44 (16)	4 (16)
**Age when first developed MNYES**, mean (SD)	31.77 (15.08)	29.89 (15.13)	-
**Clinical role as self-described**			
Cardiologist	-	1 (<1)	-
Consultant in emergency medicine	-	1 (<1)	-
Doctor	10 (2)	2 (1)	-
Gastroenterologist	-	-	1 (4)
GP	4 (1)	5 (2)	3 (12)
Occupational therapist	9 (2)	6 (2)	1 (4)
Neurologist	9 (2)	3 (1)	1 (4)
Neuropsychiatrist	1 (<1)	1 (<1)	-
Neuropsychologist	-	1 (<1)	-
Nurse	3 (1)	2 (1)	-
Physiotherapist	16 (4)	8 (3)	2 (8)
Psychiatrist	44 (10)	18 (7)	4 (16)
Psychologist	9 (2)	5 (2)	1 (4)
Psychotherapist	3 (1)	2 (1)	-
Other	8 (2)	1 (1)	-
Rheumatologist	1 (<1)	1 (<1)	
Speech and language therapist	1 (<1)	4 (1)	-
Stroke specialist	-	-	1 (4)
Assistant psychologist/Support worker	3 (1)	2 (1)	-

*14 prefer not to say **1 non-binary, Not mandatory and therefore not always provided

Figure S1 shows the proportion of original questions from each topic, which were posed by patients, caregivers, clinicians and others, who were generally individuals who met the criteria for more than one role. As can be seen, patients preferred aetiology and diagnostic questions, while clinicians emphasized treatment and outcomes. Health care services and prognosis were divided equally between patients or caregivers and clinicians.

For peer review only

Figure S1: The proportion of questions per domain suggested by patients, caregivers, clinicians and other

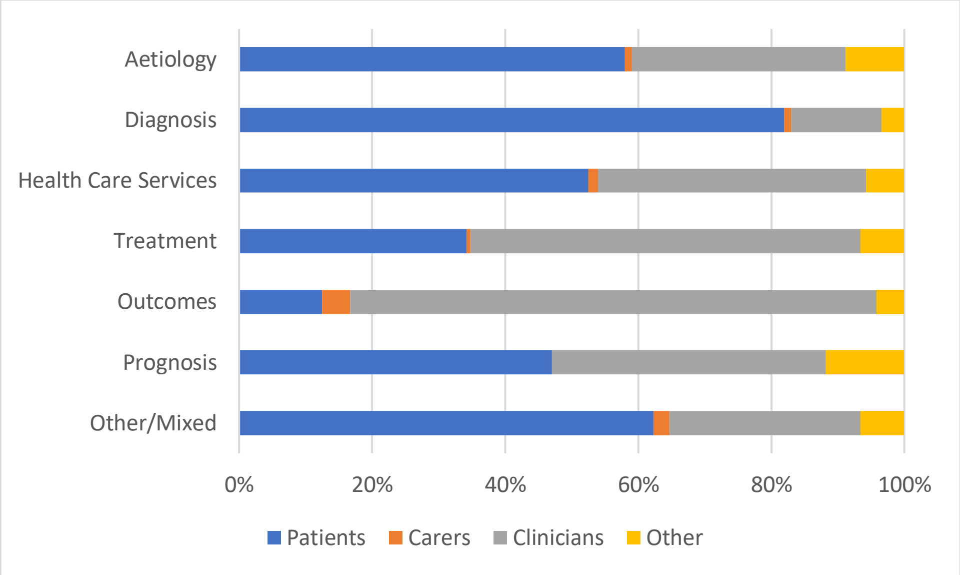

227 of the 264 (86%) people who reported gender were female and 242 of the 260 (93.1%) people who reported ethnicity, were white. The 17 resulting priorities were shortlisted for consideration at the final priority setting consensus workshop.

Box 2: research priority 11 through 17.

11. What is the relationship between mental health problems and MNYES?
12. What is the relationship over time between MNYES and known medical conditions and does that suggest some shared pathological process?
13. What is the best practice to offer optimal care for patients with MNYES?
14. What are current clinical attitudes and levels of knowledge about MNYES?
15. What are the most effective physical treatments for different symptoms of MNYES?
16. What are the most effective psychological treatments for different symptoms of MNYES?
17. Why do symptoms of MNYES fluctuate?

BMJ Open

Identifying the top research priorities in medically not yet explained symptoms (MNYES): a James Lind Alliance priority setting partnership

Journal:	*BMJ Open*
Manuscript ID	bmjopen-2022-061263.R2
Article Type:	Original research
Date Submitted by the Author:	13-Jun-2022
Complete List of Authors:	van der Feltz-Cornelis, Christina; University of York, Dept. of Health Sciences, HYMS; Tees Esk and Wear Valleys NHS Foundation Trust<br>Sweetman, Jennifer; University of York<br>Edwards, Mark; St George's University of London; St George's University Hospitals NHS Foundation Trust<br>Gall, Nicholas; University of London Kings College Hospital, Department of Cardiology<br>Gilligan, Jennifer; Tees Esk and Wear Valleys NHS Foundation Trust<br>Hayle, Steph; Patient representative<br>Kaul, Arvind; St George's University Hospitals NHS Foundation Trust<br>Moriarty, Andrew; University of York Department of Health Sciences; Hull York Medical School,<br>Perros, Petros; Royal Victoria Infirmary, Department of Endocrinology<br>Sampford, James; Tees Esk and Wear Valleys NHS Foundation Trust<br>Smith, Natalie; University of York<br>Elfeddali, Iman; Tilburg University, Tranzo Dept.; GGz Breburg, Tilburg, the Netherlands, Centre for Body Mind and Health<br>Varley, Danielle; University of York Department of Health Sciences<br>Gower, Jonathan; JLA
**Primary Subject Heading**:	Evidence based practice
Secondary Subject Heading:	Mental health
Keywords:	Adult psychiatry < PSYCHIATRY, Adult cardiology < CARDIOLOGY, Functional bowel disorders < GASTROENTEROLOGY, Rheumatology < INTERNAL MEDICINE, Adult neurology < NEUROLOGY, PRIMARY CARE

SCHOLARONE™
Manuscripts

I, the Submitting Author has the right to grant and does grant on behalf of all authors of the Work (as defined in the below author licence), an exclusive licence and/or a non-exclusive licence for contributions from authors who are: i) UK Crown employees; ii) where BMJ has agreed a CC-BY licence shall apply, and/or iii) in accordance with the terms applicable for US Federal Government officers or employees acting as part of their official duties; on a worldwide, perpetual, irrevocable, royalty-free basis to BMJ Publishing Group Ltd ("BMJ") its licensees and where the relevant Journal is co-owned by BMJ to the co-owners of the Journal, to publish the Work in this journal and any other BMJ products and to exploit all rights, as set out in our licence.

The Submitting Author accepts and understands that any supply made under these terms is made by BMJ to the Submitting Author unless you are acting as an employee on behalf of your employer or a postgraduate student of an affiliated institution which is paying any applicable article publishing charge ("APC") for Open Access articles. Where the Submitting Author wishes to make the Work available on an Open Access basis (and intends to pay the relevant APC), the terms of reuse of such Open Access shall be governed by a Creative Commons licence – details of these licences and which Creative Commons licence will apply to this Work are set out in our licence referred to above.

Other than as permitted in any relevant BMJ Author's Self Archiving Policies, I confirm this Work has not been accepted for publication elsewhere, is not being considered for publication elsewhere and does not duplicate material already published. I confirm all authors consent to publication of this Work and authorise the granting of this licence.

For peer review only

Identifying the top research priorities in medically not yet explained symptoms (MNYES): a James Lind Alliance priority setting partnership

Christina M van der Feltz-Cornelis,[1,2,3] Jennifer F Sweetman,[1] Mark Edwards,[4,5] Nicholas Gall,[6] Jennifer Gilligan,[3] Steph Hayle,[7] Arvind Kaul,[5] Andrew S Moriarty,[1,2] Petros Perros,[8] James Sampford,[3] Natalie Smith,[1] Iman Elfeddali,[9,10] Danielle Varley,[1] Jonathan Gower[11]

Corresponding author: Christina van der Feltz-Cornelis, Dept. of Health Sciences, HYMS, University of York, ARRC Building, T204, Heslington, York, United Kingdom. Phone +441904321313 Email christina.vanderfeltz-cornelis@york.ac.uk

[1] Dept of Health Sciences, University of York, York, United Kingdom

[2] Hull York Medical School, (HYMS), University of York, York, United Kingdom

[3] Tees Esk and Wear Valley NHS Foundation Trust, Darlington, United Kingdom

[4] Neuroscience Research Centre, Institute of Molecular and Clinical Sciences, St George's University of London, London, United Kingdom.

[5] St. George's University Hospitals NHS Foundation Trust, London, United Kingdom

[6] Department of Cardiology, King's College Hospital, Denmark Hill, London, SE5 9RS, United Kingdom

[7] Patient representative, North Yorkshire, United Kingdom

[8] Department of Endocrinology, Royal Victoria Infirmary, Newcastle upon Tyne, United Kingdom

[9] Tranzo Dept., Tilburg University, Tilburg, the Netherlands

[10] Centre for Body Mind and Health, GGz Breburg, Tilburg, the Netherlands

[11] James Lind Alliance, Southampton, United Kingdom

Abstract

Objectives This study establishes research priorities for Medically Not Yet Explained Symptoms, also known as Persistent Physical Symptoms (PPS) or Medically Unexplained Symptoms (MUS), from the perspective of patients, caregivers and clinicians, in a priority setting partnership (PSP) following the James Lind Alliance (JLA) approach. Research into such symptoms in general has been poorly funded over the years and so far has been primarily researcher-led with minimal input from patients, caregivers and clinicians; and sometimes has been controversial.

Design JLA priority setting partnership method. The PSP termed these symptoms Medically Not Yet Explained Symptoms (MNYES).

Methods The study was conducted according to the JLA's detailed methodology for conducting priority setting exercises. It involved five key stages: defining the appropriate term for the conditions under study by the PSP Steering Group; gathering questions on MNYES from patients, caregivers and clinicians in a publicly accessible survey; checking these research questions against existing evidence; interim prioritisation in a second survey; and a final multi-stakeholder consensus meeting to determine the top 10 unanswered research questions using the modified nominal group methodology.

Results Over 700 responses from UK patients, caregivers and clinicians were identified in the two surveys and charities contributed from a broad range of medical specialties and primary care. The final top 10 unanswered research questions cover, amongst others: treatment strategies, collaborative care pathways, training for clinicians, outcomes that matter to patients, and personalisation of treatment.

Interpretation The top 10 unanswered research questions are expected to generate much needed, relevant and impactful research into MNYES.

Word count: 3916

Key words: James Lind Alliance; Priority Setting; Medically Not Yet Explained Symptoms (MNYES); Medically Unexplained Symptoms (MUS); Persistent Physical Symptoms (PPS)

For peer review only - http://bmjopen.bmj.com/site/about/guidelines.xhtml

Strengths and limitations of this study

- Establishing research priorities for medically not yet explained symptoms (MNYES) from the perspective of patients, caregivers and clinicians for the first time is a strength of the study.

- The use of the established and transparent James Lind Alliance methodology is a strength of the study.

- Over 700 responses were gathered from patients, caregivers and clinicians from a wide range of medical specialties including primary care, indicating that the priorities were widely supported.

- Contributions of people from ethnic and gender minority groups and from underserved areas within the PSP Steering Group, surveys and final workshop supports the inclusive nature of this work and indicates these priorities are important to a diverse range of people.

- Self-descriptions of participant roles and symptoms did not always provide sufficient detail to clearly describe the variety of the participants in the sample.

For peer review only - http://bmjopen.bmj.com/site/about/guidelines.xhtml

Introduction

Medically Not Yet Explained Symptoms (MNYES), also known as Medically Unexplained Symptoms (MUS) or Persistent Physical Symptoms (PPS)(1), represent up to 30% of presentations in primary care and internal medicine settings (2-4). They can include fatigue, pain, dizziness, irritable bowel syndrome, and functional neurological symptoms (FND)(4). They are often deemed to be stress-related, or of psychological origin, and comorbid depressive or anxiety disorder occur in approximately 30% of cases (3). Patients diagnosed with these symptoms often feel that they are not taken seriously, although care may have been taken to explain their condition properly. It can take a long time to reach the conclusion that patients have MNYES; during this time they typically experience high levels of disability and face repeating appointments and diagnostic procedures. They hear that no cause can be found for their symptoms and this is often delivered by clinicians who have a dualistic view of health and disease. Disability and absenteeism occurs frequently even in patients who present only within primary care with a low number of symptoms and where the effect of demographic factores, anxiety and depressive disorder are taken into account(5-8). This inevitably leads to disappointment and frustration (9). Many clinicians lack confidence in the assessment and management of MNYES, or may exhibit behaviours perceived as dismissive. Patients often perceive a stigmatising attitude from clinicians and a sense that they are being judged as neurotic or mentally unwell (10-12). Moreover, management plans may not be sufficiently holistic to address all patient concerns, and effective treatments are scarce. All the above impact negatively on long-term prognosis.

The focus of research on MNYES is often on particular subsets of symptoms, such as chronic pain, chronic fatigue, irritable bowel syndrome, and dizziness, but lacks a comprehensive view. This has ramifications for patients who visit different clinics for their various symptoms, without sustained improvement, and as such experience unmet needs (13, 14). To address this, the University of York through the lead author (CFC) established a Priority Setting Partnership (PSP) for research needed to address MNYES. We engaged with members of the public, patients with MYNES and their caregivers, clinicians of all medical specialties known to have patients with MNYES (15), and other key stakeholders such as charities and the Royal

For peer review only - http://bmjopen.bmj.com/site/about/guidelines.xhtml

College of Psychiatry Liaison Faculty. Close collaboration with the James Lind Alliance (JLA) enabled this PSP to follow their established, rigorous approach to identify the treatment and management priorities of stakeholders (patients, caregivers, clinicians and support organisations) and to incorporate these into a research agenda (16).

The European Association of Psychosomatic Medicine has published a research agenda in this domain with one of the research priorities being patient preferences for research in this field (17). Until now, however, there has been relatively little support available for people with MNYES and those who care for them, to enable them in setting up the research agenda. Engaging patients in the research process incorporates their perspective as 'experts' from their unique experience of living with symptoms, as well as their personal knowledge regarding diagnostic trajectories and treatments in the healthcare setting if such symptoms remain (partially) unexplained (18). This study aims to address this knowledge gap.

The aim of this PSP was therefore to develop a research agenda with the joint perspectives of patients, caregivers, clinicians and support organisations across the United Kingdom as the frame of reference, to identify the most important unanswered research questions in MNYES.

Methods

This study was undertaken according to the JLA's method for undertaking PSPs as delineated in the JLAs Guidebook (16). An independent JLA Adviser (JG) guided the study team through the project and ensured that every step followed the JLA's methodology and adhered to the JLA's principles of transparency and balanced inclusion of patients, caregivers and clinicians. All materials related to this PSP can be found on the JLA website (19).

Establishing the Steering Group

In March 2020 the MNYES PSP Steering Group met for the first time. The remit of the Steering Group was to oversee, project manage and publicise the PSP, networking with charitable, patient and professional organisations to maximise the response to the surveys. The Steering Group ensured that the JLA's methodology and principles were adhered to and had no influence on the choice and ranking of the research priorities which were solely determined by the survey responses and final priority setting workshop.

For peer review only - http://bmjopen.bmj.com/site/about/guidelines.xhtml

Members of the Steering Group were selected by a snowballing method via clinics and supporting organisations, inviting clinicians providing diagnosis and treatment of the different conditions potentially covered by MNYES. Also, charities, patient networks and PPI networks were approached to recruit patients and caregivers. They were invited and selected based upon the capability, motivation and consent to contribute to the JLA PSP working group standards of reference as described in the JLA website. Efforts were made to have a representation of patients with pain, fatigue, FND, IBS and dizziness, as they are the most common MNYES conditions as shown in the literature (20). Efforts were made to include people from areas outside of London, including rural areas and underserved areas as delineated by clinics and GPs in the North of England in the Steering Group. The Steering Group was tasked with overseeing the Priority Setting Partnership by having meetings every six weeks, chaired by the JLA advisor, and making critical decisions at key points of the project (19). The composition of the Steering Group is shown in Box 1 (Supplementary File 1).

Terminology

Many terms are used for these symptoms, including, but not limited to, Persistent Somatic Symptoms (PPS) (1), Somatic Symptom and Related Disorders (SSRD)(21), Bodily Distress Disorders (BDD) (22), Medically Unexplained Symptoms (MUS), Functional symptoms, and Functional Neurological Disorder (FND). There is an ongoing debate amongst researchers and clinicians about how to refer to these conditions. Many of such terms have been deemed unsatisfactory by patients, caregivers and clinicians as well as researchers for a variety of reasons, leading to ongoing efforts from researchers to find a better term; (23-25) however, so far the patient, carer and clinician perspective regarding the choice of preferred term has been lacking. This may seem semantic, however it underpins the conceptual confusion that exists regarding these symptoms (26). Unfortunately, in some cases such uncertainty can give rise to deeply rooted controversy that ultimately can be traced back to lack of knowledge regarding the underlying conditions, and to related stigma. This knowledge gap could either be a factual lack of evidence, or a lack of availability of existing knowledge to clinicians, patients and the general public alike. Therefore, the study's PSP Steering Group took time to decide what terminology to use in the study.

A common concern appeared to be the distress caused to patients, caregivers and clinicians alike by the lack of adequate explanations, diagnostic methods and treatments for these

For peer review only - http://bmjopen.bmj.com/site/about/guidelines.xhtml

symptoms – which are often poorly understood across these groups too. This was felt to have a negative impact on clinical work and research pertaining to these conditions and to stigmatise them at a societal level. After deliberation, the PSP Steering Group agreed to use the term *Medically Not Yet Explained Symptoms (MNYES)* to describe the subject matter for the duration of the study. This was an operational definition not intended to add to or replace other definitions already in use, that was constructed to embrace the views of all stakeholders. MNYES was meant to indicate that although some insights might exist, our understanding is still incomplete. This could pertain to biological, psychological and social factors, as well as factors involving the trajectory of patients through various healthcare settings. In that sense, the choice of the term MNYES conveys a message of hope, which feeds into the effort to identify research priorities for the condition.

Inclusion and exclusion criteria

The PSP's Steering Group agreed that the remit should include the aetiology, diagnosis and treatment or medical care of patients with MNYES in the UK, as well as the organisation of services, social consequences and long term outcomes including cost implications for patients. Confirmed topics included (but were not limited to): pain, fatigue, dizziness, functional neurological disorder, bowel symptoms, palpitations and syncope. Ages 16 and older were included. Although fatigue as a symptom was considered for inclusion, Chronic Fatigue Syndrome was considered out of scope since there was another PSP addressing this.

Patient and public involvement

A core principle of JLA priority setting partnerships is collaboration between all stakeholders (patients, caregivers, and clinicians) to ensure their views are represented at each stage of the process. At the level of the steering group, patients, caregivers and clinicians were members of the MNYES Steering Group, represented at every meeting, and involved in the development of PSP surveys. They were involved in the organisation of uncertainties, the wording of summary questions, and the verification of evidence checking. At the level of the surveys, patients, public and supporting organisations participated in the surveys as shown in Table 1 (Supplementary File 1). The final workshop also included patients, caregivers and clinicians in the final prioritisation process to establish the top 10 research priorities for MNYES. Furthermore, there were observers representing supporting organisations and relevant

For peer review only - http://bmjopen.bmj.com/site/about/guidelines.xhtml

charitable organisations during the final workshop. All Steering Group members were invited to contribute to the dissemination of the surveys; the information shared by this PSP was developed with members from all stakeholder groups. All PSP steering group members were invited to contribute to the article describing the findings and one of them indeed contributed as a co-author.

Results

The process is shown in the project flowdiagram presented in Figure 1 below.

- Insert **Figure 1. Flow diagram of MNYES question prioritisation processes** –

First survey

The initial survey (June 2020 - January 2021) asked patients, caregivers and healthcare professionals to indicate their priorities for future research related to MNYES (27). There were 705 respondents who accessed the initial survey; 443 provided at least one question or statement. Respondents were 77% female, 86% white. Data from the 2011 census show that 51% of the England and Wales population were female (28) and 86% of the same population were White (29). 68% of the participants were patients or caregivers as reported in Table 2 (Supplementary File 1).

The information specialists (DV and JS) and PSP lead (CFC), grouped similar or duplicate questions into 5 domains, generating 96 draft summary questions on aetiology, diagnosis, health care services, treatment, outcomes, prognosis, and other. Those 96 draft questions were reviewed by small groups of PSP Steering Group members that comprised clinicians, patients and caregivers. Further consolidations were made resulting in 46 summary questions which were reviewed again and signed off at a meeting of the whole PSP Steering Group. A document illustrating this is available on the JLA website (30). Of these 46 questions, 22% related to aetiology, 24% to health and clinical services, 15% to diagnosis, 24% to the treatment of MNYES, and 15% to outcomes. The proportion of questions posed by stakeholder groups, organised by topic, is shown in Figure S1 (Supplementary File 1).

For peer review only - http://bmjopen.bmj.com/site/about/guidelines.xhtml

Evidence check

The 46 summary questions were checked against published systematic reviews and clinical guidelines. We found that none of the 46 summary questions had been fully answered by previous research; some questions had been answered for specific symptoms, but not comprehensively across all MNYES symptoms. At a subsequent meeting, the Steering Group reviewed the 46 summary questions in relation to the original questions and statements from which they derived. This resulted in minor changes to the wording of these 46 questions which were then included in the interim prioritisation survey.

Interim survey

This online survey was completed by 270 participants from across the UK. Patients and caregivers made up 74% of the participants. Demographic information is shown in Table 2 (Supplementary File 1).

Final priority setting workshop

The final priority setting workshop was conducted remotely over two days. In total, 25 people participated in the workshop sessions; four JLA advisors facilitated the subgroups, eight people observed and one person provided technical support. Participants included 11 people with MNYES or caregivers, and 14 healthcare professionals representing psychiatry, general practice, stroke, neurology, physiotherapy, psychology, occupational therapy and gastroenterology. The final top 10 research priorities were agreed by consensus between all the participants as listed in Figure 2. They were placed on the James Lind Alliance Website (31).

- Insert **Figure 2. Top 10 research priorities** -

The research priorities which were ranked 11 – 17 are also listed on the JLA website (31) and in Box 2 (Supplementary File 1).

Discussion

Summary of the results

In this study, we used the JLA Priority Setting Partnership processes to identify the top 10 unanswered research questions for MNYES. We utilised the collective perspectives of patients,

For peer review only - http://bmjopen.bmj.com/site/about/guidelines.xhtml

caregivers, and clinicians and focused on areas where up-to-date, reliable research evidence is lacking. This process was supported by charitable and professional organisations across the United Kingdom. The study highlighted the paucity of evidence-driven practice in MNYES care since none of the 46 research questions gathered from our survey have been previously answered by level I evidence. Based on the extensive discussions during the meetings, the following major themes emerged from the top 10 unanswered research questions.

Theme 1: Treatment

What are the most effective treatment strategies for different symptoms of MNYES?

How can the most appropriate treatment be selected, dependent on different MNYES symptoms, that a person with MNYES is most likely to benefit from?

This pertains to all potential treatment strategies (for example: pharmacological, psychological, physical, or collaboration models) to help manage or alleviate any MNYES or combination of symptoms, such as pain, fatigue, dizziness, functional neurological disorder, bowel symptoms, palpitations and syncope. It also focuses on the best ways to match people who experience specific MNYES with the treatments that are most likely to benefit them, personalising treatment based on diagnostic factors such as a history of trauma, biomarkers or treatment needs.

Given the high unmet clinical need and the heterogeneity of MNYES, it is no surprise that this is considered an important research priority. Interestingly, treatment and personalised treatment were also research priority identified by the Delphi study conducted amongst experts in the field on behalf of the European Association of Psychosomatic Medicine (17); they can therefore be considered widely supported research priorities in this field.

Theme 2: The Role of Clinicians

How can clinicians collaborate effectively to form the most appropriate care pathway and service model to offer assessment and treatment for patients with MNYES?

What are the most effective methods for training clinicians to diagnose and treat their patients with MNYES with compassion, empathy and respect?

What are the most effective ways to support patients with MNYES and their carers to live with their symptoms?

What strategies are effective at promoting awareness and up to date clinical knowledge about MNYES amongst healthcare professionals?

For peer review only - http://bmjopen.bmj.com/site/about/guidelines.xhtml

Four of the ten research priorities involve the role of the clinicians in the diagnostic and treatment process, an indicator of the high relevance of this theme. Many different clinicians provide diagnostic assessments to people with MNYES, or are sought to provide treatment to them. The focus here is on finding the best ways for clinicians to collaborate, forming an appropriate care pathway to support people with MNYES. These could be psychiatric consultation models, multi-disciplinary team models, collaborative care models or other integrated care pathways. There is a focus on communication which acknowledges the perspective and concerns of the person experiencing MNYES. Another priority focuses on identifying options for supporting people with MNYES and their caregivers, such as for example shared decision making regarding treatment options; coping with symptoms; and rehabilitation approaches. Another priority emphasises strategies to consistently and effectively ensure that clinicians know the most up-to-date information about MNYES and let care reflect current evidence. Given the existing knowledge gaps, this is considered an important priority.

Theme 3: Symptoms and Outcomes

What outcomes matter most to patients with MNYES?

What symptoms are commonly reported by people with MNYES and what links them?

What factors affect outcomes for MNYES?

Some research priorities mention the patient perspective explicitly. Based upon the survey answers, outcomes relevant for patients may include but are not limited to: symptom reduction, changes in biomarkers; improvements in abilities to undertake daily tasks; improvements in quality of life; individual goal achievements; or improvements in functioning. The list of MNYES is extensive, and people who experience these symptoms often report living with multiple MNYES. One priority aims to identify the most commonly co-occurring symptoms and their underlying factors and mechanisms. Given the number of questions that pertained to aetiological factors and the fact that the related uncertainty plays a role in the choice of MNYES as a term, this can be considered an important research theme. Factors affecting outcome should include biomarkers, psychological factors, health services, how information is shared between clinicians and people experiencing MNYES, and social factors such as poverty, education, family dysfunction or domestic abuse, trauma and work environment.

For peer review only - http://bmjopen.bmj.com/site/about/guidelines.xhtml

Theme 4: Recovery

Which self-management techniques are effective in MNYES?

This priority concerns the strategies that people experiencing MNYES can use separately from clinic visits. The focus is to identify the most effective self-administered therapies for managing or alleviating MNYES, used separately or in combination with formal treatment. Examples of self-management approaches include education, exercise, and dietary changes. It should be noted that this research priority, in contrast to ones covered by the other themes, considers that recovery in MNYES can occur, either by recovery of symptoms or by personal recovery with ongoing symptomatology. Recovery of symptomatology is referred to as clinical recovery and is covered by the other themes. Recovery whilst symptoms are ongoing is called personal recovery (32), meaning that despite symptoms being present, the function has to some extent been restored through treatment, self-management or disability management.

In mental health research and clinical practice, especially concerning psychotic conditions, personal recovery is a construct that has increasingly gained attention over the past thirty years; however, the term has not been used in MNYES. Generally, both in clinical practice and in research, the emphasis seems to have been to either attempt to attain clinical recovery or send the patient home with the message that MNYES cannot be cured and that one would have to live with the condition. This dichotomy has fed into the ongoing controversy about how to approach MNYES. This polarising stance is unhelpful. It could provide an essential contribution to further research development in this domain, alongside the research priorities summarised in the other themes. Developing this research priority would require embracing the concept that personal recovery refers to an individual process of adaptation and development where one does not simply return to but instead grows beyond the premorbid self (33), emphasising the patient perspective.

Strengths of the study

This is the first study establishing research priorities for MNYES from the perspective of patients, caregivers and clinicians. The study follows the JLA method which offers a unique, and internationally highly regarded, approach to setting research priorities through an equal partnership between patients, carers and healthcare professionals. The priorities represent a "snapshot in time" of the areas which matter the most to patient, caregivers and clinicians. It is reproducible (the Handbook and all relevant materials are available on the JLA website for

For peer review only - http://bmjopen.bmj.com/site/about/guidelines.xhtml

this purpose) and the method can be used to "refresh" priorities at a future date to reflect changes in the management of the condition and patient/carer experiences. The use of this established and transparent James Lind Alliance methodology supports the generalisability of the results and is a strength of this study.

This is a highly contentious area; however, the research priorities were widely supported by over 400 participants including clinicians from a variety of disciplines, patients with a range of symptoms, caregivers, charitible organisations, and other supporting organisations Over 700 responses were gathered from patients, caregivers and clinicians from an array of medical specialties including primary care, indicating that the priorities were widely supported. Contributions of people from ethnic and gender minority groups and from underserved areas within the PSP Steering Group, surveys and final workshop supports the inclusive nature of this work and indicates these priorities are important to a diverse range of people.

The themes identified in this PSP cover a broad range of ideas, issues and uncertainties; these are outlined in the top 10 research priorities and also reflected in the seven research priorities that did not make the top 10. Research priorities 11, 12 and 17 would link well with theme 3 in exploring associations of MNYES with mental health and somatic comorbidity, as well as the development of symptoms over time. Priorities 13 and 14 would fit in theme two, the role of clinicians; 15 and 16 link with theme one, treatment. This suggests that the themes covered by the top 10 priorities are consistent with the other research priorities which were proposed during this priority setting process.

Limitations of the study

When comparing the participants of survey 1 with survey 2, there were 443 participants in survey 1, and 270 in survey 2. The final workshop was attended by 25 people. These are high numbers and certainly adequate for priority setting according to the JLA method. However, as the description of the roles is self-described, the variety of investigative participants remains unclear in some respects. For example, it should be pointed out that in Table 2, 10 people self described as "doctor," and 8 as "other" clinician and they may well have been doctors working in primary care as GPs, or rheumatologists; however, we do not know for sure. Regarding the patients, they would state their self-described main symptom as "pain" in approximately half of the cases; from their answers to the open questions, it emerged that this often would refer

For peer review only - http://bmjopen.bmj.com/site/about/guidelines.xhtml

to musculo-skeletal or rheumatological pain. So, while the exact variety is uncertain, however, it is unlikely that this contributed to priorities in the final list of issues related to MNYES.

The study provides a good overview of research priorities for MYNES in the UK, however given the specific cultural aspects and health care organisation in the UK, the findings may not be generalisable to other countries. A similar PSP is currently being conducted in the Netherlands and may shed light on research priorities in a non-NHS healthcare setting. This will provide an opportunity to compare and evaluate the generalisability of these findings and the influence of different cultural and healthcare settings. Future research highlighting the situation in Low and Middle Income Countries would be beneficial. The results of this PSP will enable funders to prioritise research in MNYES as outlined here and hopefully will provide new, much needed knowledge in this domain.

Conclusion

MYNES are common and reflect a high level of unmet clinical need. Incorporating patient-driven research in MNYES research can allow researchers to better address the complex care needs of people with MNYES. The most important aspect of this priority setting exercise was strengthening the relationship between patients, caregivers, clinicians and support organisations and generating a list of priorities valued by these stakeholders, which we hope will guide future research.

We have identified the top 10 research priorities in MNYES using the rigorous JLA priority setting method that may open the door to further research addressing the knowledge gaps and controversies in this area, and hopefully alleviate some of the stigma related to these conditions and the people suffering from MNYES. Identification of these top 10 research priorities is an important first step to generating relevant, impactful research that will ultimately improve the lives of people with MNYES.

Acknowledgements: The Steering Group would like to thank to all the patients, caregivers, families, friends, healthcare professionals and supporting organisations who contributed to

For peer review only - http://bmjopen.bmj.com/site/about/guidelines.xhtml

this work. In addition to the co-authors, Philippa Bolton, Sally Brabyn, Tracey Cunningham, Rosie Evans, Miriam Lomas, Margot Metz, Chris Price, Annie Shaw, Scott Spain, Lesley Spain were members of the PSP Steering Group. The following people who attended the final workshop for priority setting agreed to be named: Anna Burneika, Kit Byatt, Phoebe Cole, Tracey Cunningham, Mark Edwards, Rosie Evans, Eve Fazakerley, Jennifer Gilligan, Stephanie Johnston, Claire Jones, Hilary Lewis, Joseph Littlewood, Miriam Lomas, Andrew Moriarty, Elizabeth Paul, Emma Reinhold, Keziah Reiss, James Sampford, Annie Shaw, Gemma Smith, Martin Veysey, Juliet Wilson, Jennifer Wilson.

Ethics statement: Ethical approval was not required as confirmed by the research governance board of the Department of Health Sciences of the University of York.

Data availability statement

Data relevant to the study are included in the article or on the James Lind Alliance website (https://www.jla.nihr.ac.uk/priority-setting-partnerships/medically-not-yet-explained-symptoms/) and linked University of York website (https://www.york.ac.uk/healthsciences/research/mental-health/projects/mnyes/).

Conflicts of interest statement

The authors have no conflicts of interest to declare.

Author statement

CFC was the project lead, instigating the application to the JLA. CFC, NS, JFS and JG organised the Steering Group meetings. JFS and DV designed and built the surveys, analysed the data, and conducted the evidence check under supervision of JG and CFC. CFC, JFS, and NS wrote the majority of the final manuscript. JG (JLA Chair) chaired all the meetings, led the consensus workshop and ensured compliance with methodology throughout. NS and JFS took minutes for Steering Group meetings, built and distributed surveys, and organised the consensus workshop. JFS conducted the searches for the evidence check and screened the results with CFC. Members of the Steering Group CFC, JFS, ME, NG, JG, SH, AK, ASM, PP, JS, NS, IE, DV, PB, SB, TC, RE, ML, MM, CP, AS, SS, LS all attended a majority of the meetings, agreed the initial protocol and the evidence check protocol, piloted and signed off the surveys and disseminated

For peer review only - http://bmjopen.bmj.com/site/about/guidelines.xhtml

them, checked the raw questions against the indicative ones, reviewed the evidence check results and agreed the final longlist. TC, ME, JG, ML, ASM, JFS, and AS were present at the final consensus workshop. All authors reviewed and contributed to the final manuscript and approved it prior to submission.

Funding

This work was part-funded by the Wellcome Trust [ref: 204829] from the overall Wellcome Trust ISSF award through the Centre for Future Health (CFH) at the University of York.

For peer review only

References

1. Patel M, James K, Moss-Morris R, Husain M, Ashworth M, Frank P, Ferreira N, Mosweu I, McCrone P, Hotopf M, David A, Landau S, Chalder T. Persistent physical symptoms reduction intervention: a system change and evaluation (PRINCE)-integrated GP care for persistent physical symptoms: protocol for a feasibility and cluster randomised waiting list, controlled trial. BMJ Open. 2019 Jul 23;9(7):e025513. doi: 10.1136/bmjopen-2018-025513.
2. Hoedeman R, Blankenstein AH, van der Feltz-Cornelis CM, Krol B, Stewart R, Groothoff JW. Consultation letters for medically unexplained physical symptoms in primary care. Cochrane Database of Systematic Reviews. 2010(12).
3. Van Hemert AM, Hengeveld MW, Bolk JH, Rooijmans HG, Vandenbroucke JP. Psychiatric disorders in relation to medical illness among patients of a general medical out-patient clinic. Psychological medicine. 1993;23(1):167-73.
4. Kirmayer LJ, Groleau D, Looper KJ, Dao MD. Explaining medically unexplained symptoms. The Canadian journal of psychiatry. 2004;49(10):663-72.
5. van der Leeuw G, Gerrits MJ, Terluin B, Numans ME, van der Feltz-Cornelis CM, van der Horst HE, Penninx BW, van Marwijk HW. The association between somatization and disability in primary care patients. J Psychosom Res. 2015 Aug;79(2):117-22. doi: 10.1016/j.jpsychores.2015.03.001. Epub 2015 Mar 11. PMID: 25824596.
6. den Boeft M, Twisk JW, Hoekstra T, Terluin B, Penninx BW, van der Wouden JC, Numans ME, van der Horst HE. Medically unexplained physical symptoms and work functioning over 2 years: their association and the influence of depressive and anxiety disorders and job characteristics. BMC Fam Pract. 2016 Apr 14;17:46. doi: 10.1186/s12875-016-0443-x. PMID: 27079909; PMCID: PMC4831095.
7. Hoedeman R, Krol B, Blankenstein N, Koopmans PC, Groothoff JW. Severe MUPS in a sick-listed population: a cross-sectional study on prevalence, recognition, psychiatric co-morbidity and impairment. BMC Public Health. 2009 Dec 1;9:440. doi: 10.1186/1471-2458-9-440. PMID: 19951415; PMCID: PMC2793259.
8. Escobar JI, Waitzkin H, Silver RC, Gara M, Holman A. Abridged somatization: a study in primary care. Psychosom Med. 1998 Jul-Aug;60(4):466-72. doi: 10.1097/00006842-199807000-00012. PMID: 9710292.
9. Salmon P, Peters S, Stanley I. Patients' perceptions of medical explanations for somatisation disorders: qualitative analysis. BmJ. 1999;318(7180):372-6.
10. Tolchin B, Tolchin DW, Stein MA. How Should Clinicians Minimize Harms and Maximize Benefits When Diagnosing and Treating Disorders Without Biomarkers? AMA journal of ethics. 2021;23(7):530-6.
11. Hatcher S, Arroll B. Assessment and management of medically unexplained symptoms. Bmj. 2008;336(7653):1124-8.
12. Stone J, Wojcik W, Durrance D, Carson A, Lewis S, MacKenzie L, et al. What should we say to patients with symptoms unexplained by disease? The "number needed to offend". Bmj. 2002;325(7378):1449-50.
13. Carson AJ, Ringbauer B, Stone J, McKenzie L, Warlow C, Sharpe M. Do medically unexplained symptoms matter? A prospective cohort study of 300 new referrals to neurology outpatient clinics. Journal of Neurology, Neurosurgery & Psychiatry. 2000;68(2):207-10.

For peer review only - http://bmjopen.bmj.com/site/about/guidelines.xhtml

14. Reid S, Wessely S, Crayford T, Hotopf M. Frequent attenders with medically unexplained symptoms: service use and costs in secondary care. The British Journal of Psychiatry. 2002;180(3):248-53.
15. Nimnuan C, Hotopf M, Wessely S. Medically unexplained symptoms: an epidemiological study in seven specialities. Journal of psychosomatic research. 2001;51(1):361-7.
16. James Lind Alliance. The James Lind Alliance Guidebook Version 7. 2018.
17. van der Feltz-Cornelis CM, Elfeddali I, Werneke U, Malt UF, Van den Bergh O, Schaefert R, et al. A European research agenda for somatic symptom disorders, bodily distress disorders, and functional disorders: results of an estimate-talk-estimate Delphi expert study. Frontiers in psychiatry. 2018;9:151.
18. Gill PJ, Cartwright E. Partnering with patients in the production of evidence. Royal Society of Medicine; 2021.
19. James Lind Alliance. Priority Setting Partnership: Medically Not Yet Explained Symptoms 2021 [Available from: https://www.jla.nihr.ac.uk/priority-setting-partnerships/medically-not-yet-explained-symptoms/.
20. Kellner R & Sheffield BF. The one-week prevalence of symptoms in neurotic patients and normals. Am J Psychiatry 1973;50:734-41
21. American Psychiatric Association. Diagnostic and statistical manual of mental disorders: DSM-5. Arlington, VA. 2013.
22. World Health Organization. International Classification of Diseases-11 (ICD-11)2019.
23. van der Feltz-Cornelis CM, Elfeddali I, Werneke U, Malt UF, Omer Van den Bergh O, Schaefert R, Kop WJ, Lobo A, Sharpe M, Söllner W, Löwe B. A European Research Agenda for Somatic Symptom Disorders, Bodily Distress Disorders, and Functional Disorders: Results of an Estimate-Talk-Estimate Delphi Expert Study. Front Psychiarty 2018 May 14;9:151. DOI: 10.3389/fpsyt.2018.00151
24. Ding JM, Kanaan RA. What should we say to patients with unexplained neurological symptoms? How explanation affects offence. J Psychosom Res. 2016 Dec;91:55-60. doi: 10.1016/j.jpsychores.2016.10.012.
25. Stone J, Wojcik W, Durrance D, Carson A, Lewis S, MacKenzie L, Warlow CP, Sharpe M. What should we say to patients with symptoms unexplained by disease? The "number needed to offend". BMJ. 2002 Dec 21;325(7378):1449-50.
26. Van der Feltz-Cornelis C, Van Dyck R. The notion of somatization: an artefact of the conceptualization of body and mind. Psychotherapy and psychosomatics. 1997;66(3):117-27.
27. University of York. Research priority setting for Medically Not Yet Explained Symptoms (MNYES) in an Anglo-Dutch partnership with the James Lind Alliance 2021 [Available from: https://www.york.ac.uk/healthsciences/research/mental-health/projects/mnyes/.
28. GOV.UK. Male and female populations 2018 [Available from: https://www.ethnicity-facts-figures.service.gov.uk/uk-population-by-ethnicity/demographics/male-and-female-populations/latest.
29. GOV.UK. Population of England and Wales 2018 [Available from: https://www.ethnicity-facts-figures.service.gov.uk/uk-population-by-ethnicity/national-and-regional-populations/population-of-england-and-wales/latest.
30. https://www.jla.nihr.ac.uk/priority-setting-partnerships/medically-not-yet-explained-symptoms/downloads/medically-not-yet-explained-symptoms-psp-all-questions-data.doc

For peer review only - http://bmjopen.bmj.com/site/about/guidelines.xhtml

31. James Lind Alliance. Medically Not Yet Explained Symptoms Top 10 priorities 2021 [Available from: https://www.jla.nihr.ac.uk/priority-setting-partnerships/medically-not-yet-explained-symptoms/top-10-priorities.htm.

32. Slade M, Amering M, Oades L. Recovery: an international perspective. Epidemiology and Psychiatric Sciences. 2008;17(2):128-37.

33. Slade M. Personal recovery and mental illness: A guide for mental health professionals: Cambridge University Press; 2009.

For peer review only

For peer review only - http://bmjopen.bmj.com/site/about/guidelines.xhtml

Figure 1: Flow diagram of MNYES question prioritisation processes

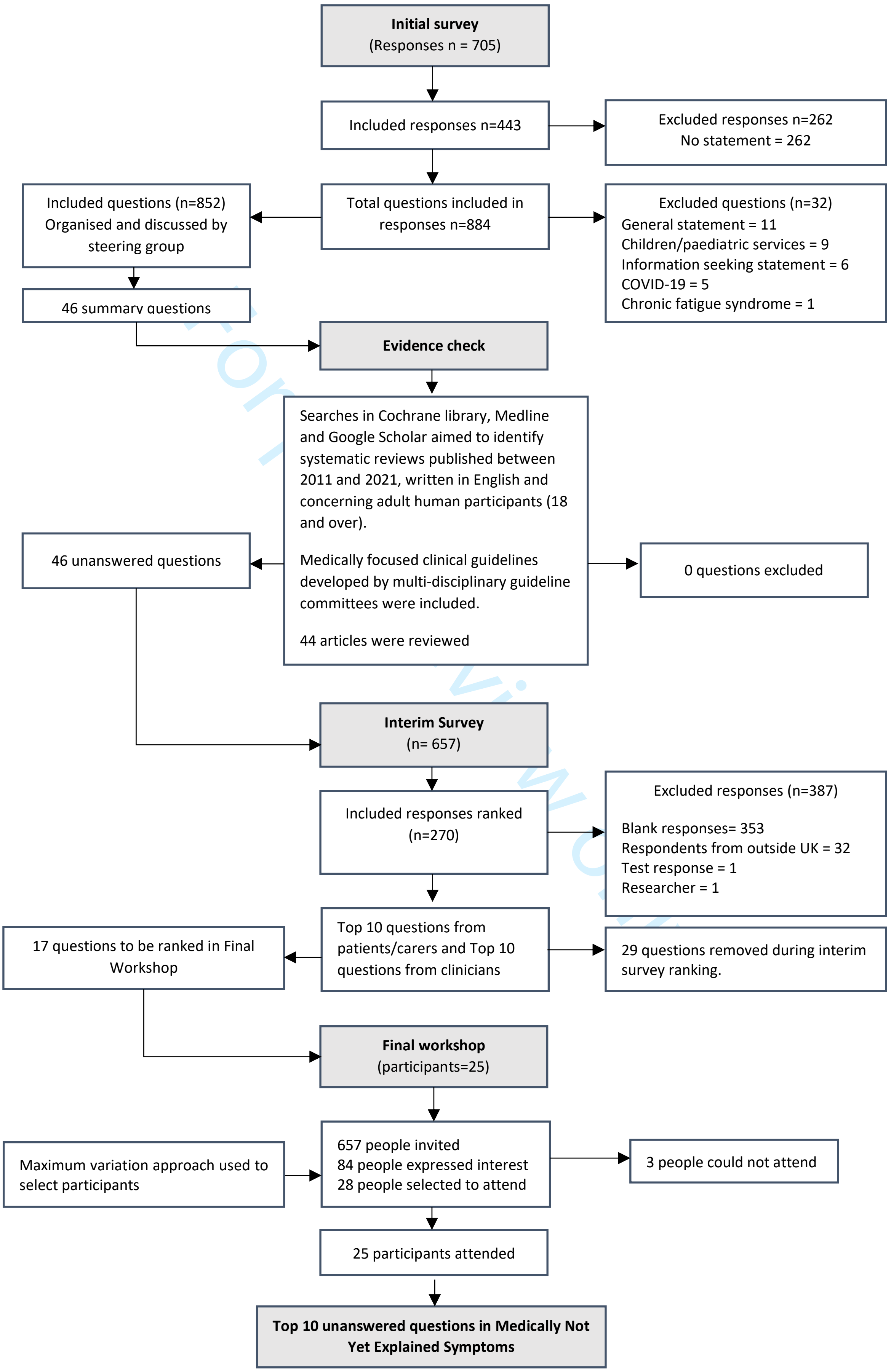

Top-10 Research Priorities
Medically Not Yet Explained Symptoms (MNYES)

1. What are the most effective treatment strategies for different symptoms of MNYES?
2. How can clinicians collaborate effectively to form the most appropriate care pathway and service model to offer assessment and treatment for patients with MNYES?
3. What are the most effective methods for training clinicians to diagnose and treat their patients with MNYES with compassion, empathy and respect?
4. What outcomes matter most to patients with MNYES?
5. What are the most effective ways to support patients with MNYES and their carers to live with their symptoms?
6. How can the most appropriate treatment be selected, dependent on different MNYES symptoms, that a person with MNYES is most likely to benefit from?
7. What symptoms are commonly reported by people with MNYES and what links them?
8. What factors affect outcomes for MNYES?
9. What strategies are effective at promoting awareness and up to date clinical knowledge about MNYES amongst healthcare professionals?
10. Which self-management techniques are effective in MNYES?

Figure 2: Top 10 Research Priorities for Medically Not Yet Explained Symptoms

For peer review only - http://bmjopen.bmj.com/site/about/guidelines.xhtml

Appendix

Key words included in evidence check searches

**Symptom terms**	**Publication focus**
Medically not yet explained symptoms	Guideline
Medically unexplained symptoms (MUS)	Mechanism
Persistent Somatic Symptoms	Pathology
Somatic Symptom and Related Disorders (SSRD)	Development
Chronic pain	Assessment
Unexplained pain	Diagnosis
Fatigue	Investigation
Unexplained fatigue	Treatment
Conversion disorder	Management
Neurological conversion symptoms	Healthcare service
Functional neurological disorder	Patient care
Functional symptoms	Care pathway
Functional neurological symptom	Outcome
Functional cognitive symptom	Prognosis
Functional weakness	
Dissociative disorders	
Dissociative neurological disorder	
Dissociative syncope	
Unexplained dizziness	
Fibromyalgia	
Irritable bowel syndrome (IBS)	
Nonepileptic seizures	

Acquiring funding, international aspect, and establishing support by the James Lind Alliance

Funding for the study was acquired by the PSP lead (CFC) in November 2019. Part of the collaboration was envisioned to support setting up a parallel PSP Steering Group to explore the same question in the Netherlands, in order to have an international perspective. This article focuses on the description of the process in the UK. Support by the James Lind Alliance Priority Setting Partnership was secured in December 2019 and a JLA Advisor (JG) appointed to the project.

Box 1: Steering group composition

Clinicians: One general practitioner(GP); consultants in cardiology (1), neurology (1), endocrinology (1), clinical liaison psychiatry (3) and rheumatology (1) one stroke physician, a clinical psychologist and one physiotherapist.

Patients and caregivers: Four patients with a variety of pain, fatigue and functional neurological symptoms; one caregiver;

Other: Two information specialists and two project coordinators to facilitate the PSP working group. They prepared meeting documents, surveys, supported recruitment, completed evidence checking and analysis but did not engage in the priority setting.

The consultative process

The study followed several steps in order to establish research priorities, using a mix of online surveys and a priority setting workshop. We created a website to host the surveys (27) and sent out the website link for our partner organisations to distribute within their networks.

Gathering uncertainties

The initial survey was launched between June 2020 and January 2021 and asked patients, caregivers and healthcare professionals to indicate their priorities for future research related to MNYES (27). We promoted the survey through partner organisations' websites, and their social media platforms. In addition, the clinics of clinicians involved in the PSP Steering Group distributed the QR code to their patients. We sent out email reminders to members of professional associations, sent Twitter invitations and placed the survey on the University of York and JLA websites. Distribution of the survey was supported by the members of the PSP, several associations, networks, networking groups and charities. An overview of supporting

For peer review only

organisations is shown in Table 1, below. Some of them collaborated because they found that patients with unexplained symptoms often visit their websites and related specialists to assess their symptoms.

Table 1: Supporting organisations who have promoted this work

Charities	Professional networks	Other networks and Social media groups
British Thyroid Foundation	Allied Health Professional FND Networking groups	INCLUDE (University of York Disability Network)
Fibromyalgia Association UK	Clinical special interest groups	James Lind Alliance
FND Hope	Liaison psychiatry networks	People who have shared information with their friends and family
Graham Hughes International	Royal College of Psychiatrists	Individuals and organisations active on twitter
Guts UK		Special interest and support groups on facebook
Health Unlocked		
Pain UK		
POTS-UK		

We targeted patients with MNYES such as pain, bowel problems, functional neurlogical symptoms, or with diagnoses which comprise a set of symptoms which are medically unexplained such as postural orthostatic tachycardia syndrome (PoTS); their caregivers; and the clinicians who treat them, to participate. Survey respondents were asked whether they had experienced MYNES, in which case they were to select their symptoms from a list, or were healthcare professionals, in which case they were asked to state their profession. Anonymity was preserved in all cases accordance with General Data Protection regulations.

Data analysis and verifying uncertainties

We downloaded the online survey results through Qualtrics and exported them into Microsoft Excel. The responses to this survey were organised into summary questions and these were then checked to make sure that they had not already been answered by research.(16) Our health information specialists (DV and JS) and PSP lead (CFC) grouped the responses into themes and each was analysed in small groups by 3 or 4 members of the Steering Group; one member of each small group was a patient or caregiver and the other members were healthcare professionals. Summary questions were developed, which encapsulated groupings of similar responses to the survey. Responses were removed if they were outside the scope

For peer review only

of the survey or general statements which would be unanswerable by research. The outputs from the small group discussions were reviewed at a subsequent meeting of the full PSP Steering Group.

One health information specialist (JS) checked each in-scope question with existing clinical guidelines published by multi-disciplinary guideline committees and systematic reviews, to ensure that the question had not been already fully answered with high-level evidence. 44 articles were shortlisted from searches conducted in the Cochrane Library, Medline and Google Scholar; key words for the search used are included in the appendix. Reference lists of these studies were also reviewed to identify further relevant references. Articles published between 2011 and 2021 were considered relevant by the Steering Group. Each question was coded to indicate whether it was 'answered'; 'partially answered' or 'unanswered' through the research literature.

Interim priority setting

Between May and July 2021, the second online survey asked participants to select their top 10 priorities from the list of uncertainties presented (19). The second survey was launched independently from the first survey. Questions were presented in a random order to each survey participant to reduce bias. The priorities of patients and caregivers and the priorities from healthcare professionals were ranked separately. The top 10 priorities identified by patients and caregivers were then combined with the top 10 priorities from healthcare professionals to create a shortlist of 17 priorities as there were 3 overlapping priorities.

Final workshop

The 17 top ranked questions were taken forward to the final priority setting consensus meeting that took place virtually on 16th and 17th September 2021. We invited volunteers from supporting organisations, two of whom joined the meeting. We also invited individuals who took part in the online survey to participate in the meeting. 25 participants took part in the workshop comprising 10 patients with a variety of MNYES symptoms, 1 caregiver, and 14 health care professionals. The workshop participants were divided into four groups, each with a balanced mix of patients/caregivers and clinicians. Each group also had observers who did not participate in the process, to fulfill the requirement of transparency of the JLA process. Each group was asked to rank the uncertainties through group discussions using the modified

For peer review only

nominal group technique facilitated by an independent JLA Advisor. Each participant was provided with a printed and electronic copy of the different 'unanswered' questions, and the JLA Advisor leading each group shared an image containing the unanswered questions to aid the group in ranking the 17 uncertainties. The rankings from the four groups were combined and all the workshop participants then came together to discuss these rankings. Similarities and differences between the group rankings were highlighted by the JLA facilitator leading the workshop (JG) and participants were invited to comment on the initial combined ranking. Following this, participants were reallocated into four new groups, maintaining the representation of patients/caregivers and clinicians within each group, to consider the combined group ranking from the previous session. During this session, there was a specific focus placed on the ordering of uncertainties across the top 17 unanswered questions. The rankings from each of the four groups were once again combined and were presented to the whole group for discussion. By the end of the priority setting meeting, we reached consensus on the top 10 UK research priorities for MNYES.

The respondents were 77% female, 86% white; data from the 2011 census show that 51% of the England and Wales population were female (28) and 86% of the same population were White (29). Of the 884 statements, 852 were in scope and 32 out-of-scope. The out-of-scope submissions included general statements (N = 11), children/paediatric services (N = 9), information seeking statements (N = 6), or pertaining to COVID-19 (N = 5) and chronic fatigue syndrome (N = 1).

Table 2: Participant demographic information

	**Initial Survey** (n=443)	**Interim Survey** (n=270)	**Final Workshop** (n=25)***
**Gender (%)**			
Male	89 (20)	33 (12)	6 (24)
Female	339 (77)	227 (84)	18 (72)
Other	14*(3)	2** (1)	1 (4)
**Ethnicity (%)**			
Asian/ Asian British	12 (3)	5 (2)	-
Arab	1 (<1)	-	-
Black/ Black British	3 (1)	2 (1)	-
Mixed/ Multiple	7 (2)	7 (2)	-
White	381 (86)	242 (90)	25 (100)
Other	17 (4)	4 (1)	-
**Age**, mean (SD)	47.65 (12.15)	42.55 (13.26)	39.29 (13.74)
**Experience (%)**			
Patient	288 (65)	183 (68)	10 (40)

For peer review only

Caregiver	13 (3)	17 (6)	1 (4)
Clinician	121 (27)	65 (24)	14 (56)
Other	21 (5)	5 (2)	-
**MNYES symptoms as self-described(%)**			
Pain	219 (49)	147 (54)	6 (24)
Fatigue	236 (53)	146 (54)	8 (32)
Dizziness	156 (35)	91 (34)	5 (20)
Heart palpitations/fainting	134 (30)	83 (31)	5 (20)
Stomach/bowel problems	197 (44)	119 (44)	5 (20)
Problems with movement (tremors, shakes, weakness)	133 (30)	114 (42)	6 (24)
Unexplained blackouts, seizures, sudden loss of awareness	54 (12)	60 (22)	3 (12)
Other (e.g. brain fog, eye and skin problems)	126 (28)	44 (16)	4 (16)
**Age when first developed MNYES**, mean (SD)	31.77 (15.08)	29.89 (15.13)	-
**Clinical role as self-described**			
Cardiologist	-	1 (<1)	-
Consultant in emergency medicine	-	1 (<1)	-
Doctor	10 (2)	2 (1)	-
Gastroenterologist	-	-	1 (4)
GP	4 (1)	5 (2)	3 (12)
Occupational therapist	9 (2)	6 (2)	1 (4)
Neurologist	9 (2)	3 (1)	1 (4)
Neuropsychiatrist	1 (<1)	1 (<1)	-
Neuropsychologist	-	1 (<1)	-
Nurse	3 (1)	2 (1)	-
Physiotherapist	16 (4)	8 (3)	2 (8)
Psychiatrist	44 (10)	18 (7)	4 (16)
Psychologist	9 (2)	5 (2)	1 (4)
Psychotherapist	3 (1)	2 (1)	-
Other	8 (2)	1 (1)	-
Rheumatologist	1 (<1)	1 (<1)	
Speech and language therapist	1 (<1)	4 (1)	-
Stroke specialist	-	-	1 (4)
Assistant psychologist/Support worker	3 (1)	2 (1)	-

*14 prefer not to say **1 non-binary, Not mandatory and therefore not always provided

Figure S1 shows the proportion of original questions from each topic, which were posed by patients, caregivers, clinicians and others, who were generally individuals who met the criteria for more than one role. As can be seen, patients preferred aetiology and diagnostic questions, while clinicians emphasized treatment and outcomes. Health care services and prognosis were divided equally between patients or caregivers and clinicians.

For peer review only

Figure S1: The proportion of questions per domain suggested by patients, caregivers, clinicians and other

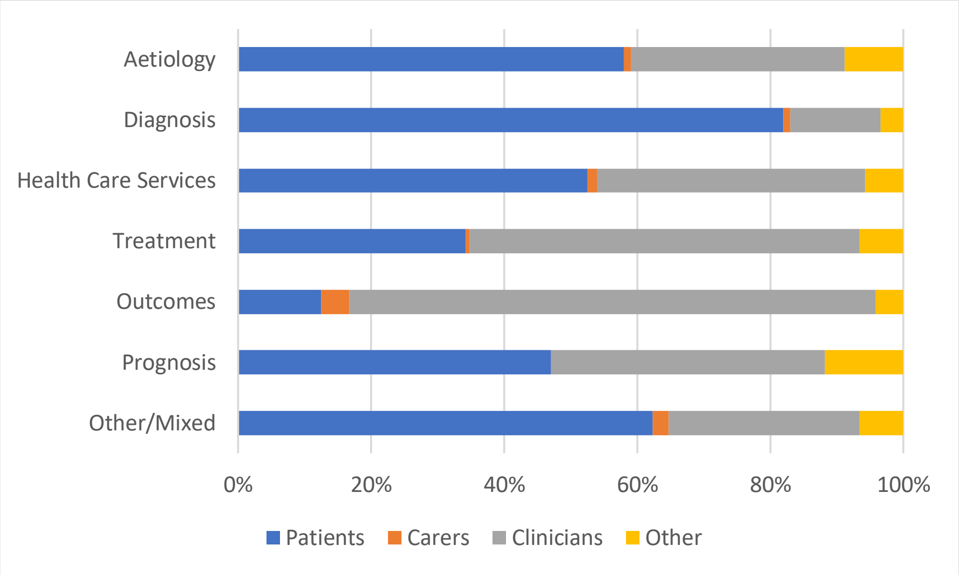

227 of the 264 (86%) people who reported gender were female and 242 of the 260 (93.1%) people who reported ethnicity, were white. The 17 resulting priorities were shortlisted for consideration at the final priority setting consensus workshop.

Box 2: research priority 11 through 17.

11. What is the relationship between mental health problems and MNYES?
12. What is the relationship over time between MNYES and known medical conditions and does that suggest some shared pathological process?
13. What is the best practice to offer optimal care for patients with MNYES?
14. What are current clinical attitudes and levels of knowledge about MNYES?
15. What are the most effective physical treatments for different symptoms of MNYES?
16. What are the most effective psychological treatments for different symptoms of MNYES?
17. Why do symptoms of MNYES fluctuate?

For peer review only
